# The impact of intensity‐modulated radiotherapy in conjunction with chemotherapy on proximal pT3N0 rectal cancer patients after total mesorectum excision

**DOI:** 10.1002/cam4.6691

**Published:** 2023-11-06

**Authors:** Chuan‐Yin Fang, Hsuan‐Yu Chen, Hsin‐Yi Yang, Yuk‐Wah Tsang, Cheng‐Yen Lee, Chih‐Chia Chang, I‐Chen Lin, Yun‐Jhong Huang, Chun‐Ting Chu, Yu‐Wen Wang

**Affiliations:** ^1^ Division of Colorectal Surgery, Department of Surgery Ditmanson Medical Foundation Chia‐Yi Christian Hospital Chiayi City Taiwan; ^2^ Institute of Statistical Science, Academia Sinica Taipei Taiwan; ^3^ Clinical Research Center Ditmanson Medical Foundation Chia‐Yi Christian Hospital Chiayi Taiwan; ^4^ Department of Radiation Oncology Ditmanson Medical Foundation Chia‐Yi Christian Hospital Chiayi Taiwan; ^5^ Division of Colorectal Surgery, Department of Surgery Antai Medical Care Coporation Antai Tian‐Shang Memorial Hospital Pingtung Taiwan

**Keywords:** adjuvant therapy, chemoradiation, radiotherapy, rectal cancer, surgery

## Abstract

**Background:**

This study aimed to ascertain if the incorporation of intensity‐modulated radiotherapy (IMRT) with chemotherapy (CTx) offered any advantages for patients diagnosed with stage pT3N0 rectal cancer located in the proximal (upper) region following a complete total mesorectum excision (TME).

**Methods:**

We retrospectively examined medical records of stage II/III rectal cancer patients who had undergone CTx or concurrent chemoradiation (CCRT) with IMRT after a successful TME. We juxtaposed a variety of survival outcomes across two patient cohorts. Each outcome was further classified according to Gunderson's risk stratification between proximal and distal (middle and low) rectal cancer patients, and we evaluated the factors associated with each outcome.

**Results:**

The median follow‐up duration was 4.9 years. Our research comprised 236 rectal adenocarcinoma patients treated at our institution between 2007 and 2019. They received either the CTx (*n* = 135) or the CCRT (*n* = 101) with 10‐year locoregional recurrence‐free survival (LRRFS) of 90.1% and 96.1%, respectively (*p* = 0.163). However, after performing multivariate adjustments, a pattern emerged hinting at a better LRRFS for the CCRT group (*p* = 0.052). Perforation had a strong correlation with locoregional recurrence. No significant differences were observed in other survival between the two treatment arms and their respective subgroups. The CCRT group witnessed significantly higher immediate and chronic complications with *p* = 0.007 and 0.009, respectively. The CCRT group had two secondary cancer‐related fatalities (2%, one attributed to IMRT), and another reported by the CTx group (1%). The sole classified locoregional recurrence within the cohort of 37 individuals treated with CTx for proximal pT3N0 rectal cancer was, in fact, the development of sigmoid colon cancer.

**Conclusion:**

The results suggest that for patients with proximal pT3N0 rectal cancer post‐TME, IMRT is better when not combined with CTx, except in highly perilous scenarios or those involving perforation.

## Graphical abstract

The 10‐year locoregional recurrence‐free survival with or without radiotherapy (RT) is essentially identical for clear‐margin proximal rectal pT3N0 cancer patients. More significant complications will be associated with RT. We suggest that for patients with proximal pT3N0 rectal cancer postoperation, RT is better when not combined with chemotherapy, except in highly perilous scenarios or those involving perforation.

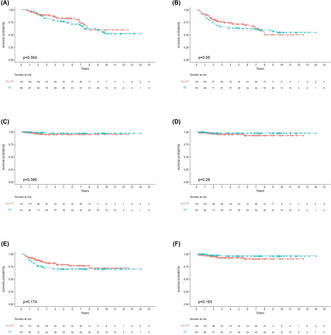

## 1 INTRODUCTION

The standard course of treatment for locally advanced rectal cancer (LARC), as suggested by the National Comprehensive Cancer Network (NCCN) guidelines, typically involves preoperative chemotherapy (CTx) and radiotherapy (RT), followed by surgery.^1^ Despite this, numerous randomized studies have been unable to demonstrate a significant improvement in overall survival (OS) or disease progression‐free survival (DFS) when combining preoperative CTx with RT as opposed to adjuvant postoperative CTx and RT. Nevertheless, these studies did consistently reveal that this treatment method led to superior rates of locoregional recurrence‐free survival (LRRFS), sphincter preservation, and a generally reduced toxicity profile (in most cases).^2^, ^3^, ^4^ Other investigations have suggested that postoperative concurrent chemoradiation (CCRT) achieves 3–5‐year OS and DFS comparable to the standard treatment. At the same time, intensity‐modulated radiotherapy (IMRT) noticeably reduces complications and side effects.^5^ Based on this evidence, upfront surgery has become a viable treatment choice for LARC patients at our hospital.

Whether or not to supplement CTx with RT after surgery is pivotal yet challenging for rectal cancer patients. Pelvic relapse rates of approximately 25% have been observed in patients showing pathological evidence of extramural tumor invasion (pT3/4) or lymph node (LN) involvement (pN+).^6^, ^7^ To boost locoregional control, adjuvant RT is generally prescribed alongside CTx for these patients.^7^ However, RT's side effects are significant, and there is an associated mortality risk due to the treatment itself.^8^, ^9^, ^10^ Total mesorectum excision (TME) surgery can provide satisfactory local control on its own,^11^, ^12^, ^13^, ^14^ and when coupled with adjuvant CTx, the results can be significantly enhanced.^15^, ^16^, ^17^ Cancers above the anterior peritoneal reflection (in the proximal or upper rectum) tend to recur less frequently, and the benefit of adjuvant RT for such patients remains debatable.^18^, ^19^ Gunderson and colleagues have stratified patients with T3/4 or N+ according to their risk levels, which are intermediate risk (pT1N1, pT2N1, and pT3N0), moderately high risk (pT3N1, pT4N0, pT1N2, and pT2N2), and high risk (pT3N2, pT4N1, and pT4N2).^16^ Some researchers have posited that it may be reasonable to exclude adjuvant RT from the treatment regimen of at least certain upper rectal pT3N0 cancer patients.^20^, ^21^, ^22^ In 2020, the NCCN guidelines introduced the option of standalone adjuvant chemotherapy for patients with margin‐negative proximal pT3N0 cancer.^1^ However, adjuvant CCRT remains an available treatment option for these patients after low anterior resection (LAR). Nonetheless, sufficient evidence must be provided to make informed decisions about the groups of patients that should receive RT. The potential benefits of tumor control must be cautiously balanced against the harmful effects of RT toxicity. Recent changes in NCCN guidelines have led to increased use of IMRT due to its potential to reduce toxicity.^1^ However, as of now, there have not been any large‐scale randomized clinical trials that assess the use of adjuvant IMRT after TME in patients with upper rectum tumors from each risk category. In this study, we aim to assess if integrating IMRT postoperative chemotherapy (CTx) regimen benefits patients with pT3N0 upper rectal cancer. We plan to employ a meticulous research approach involving a retrospective review of patient records. In addition, we will compare treatment outcomes and potential complications between both groups to evaluate the impact of IMRT. Essential aspects under examination include survival rates, recurrence rates, and side effects experienced by patients. This comprehensive approach will enable us to offer solid evidence on the merits and potential drawbacks of IMRT integration in treating pT3N0 upper rectal cancer patients, thereby aiding in informed decision‐making and enhancing treatment strategies.

## 2 METHODS

This research received the approval of the Ditmanson Medical Foundation Chia‐Yi Christian Hospital's Institutional Review Board (Approval No: IRB2020147). While we did not specifically obtain informed consent, all data used in the analysis were thoroughly anonymized.

### 2.1 Study cohort

We gathered image and medical records data for patients with rectal cancer, free from distant metastasis, diagnosed at our institution between January 2007 and December 2019. All the patients were pathologically confirmed to have stage T3/4 or N+ adenocarcinoma and had undergone TME surgery. Our study's inclusion criteria were as follows: patients aged 20 or older with good performance scores (Eastern Cooperative Oncology Group [ECOG] ≤3); diagnosed as pathologically stage II/III; and had sufficient circumferential resection margin (CRM) (≥1 mm), distal margin (≥1 cm), and retrieved ≥12 LNs. Exclusion criteria included: a secondary cancer diagnosis concurrent with or before the rectal cancer diagnosis, tumors situated 16 cm or more above the anal verge (AAV); tumors in the sigmoid–rectal junction without definitive rectal invasion as determined by imaging studies or intraoperatively; a history of significant pelvic surgery or radiation therapy (RT); administration of an unclear RT regimen at doses between 18 and 36 Gy; the final RT date being >180 days postsurgery; and suspected recurrence before RT initiation, as suggested by postoperative images.

The patient's primary physicians were responsible for determining treatment, with most patients under the surveillance and review of a tumor team, which included colorectal surgeons, medical oncologists, diagnostic radiologists, nuclear medicine physicians, and radiation oncologists. Pretreatment evaluations consisted of physical examination, fiberoptic colonoscopy, contrast‐enhanced computed tomography (CT), or magnetic resonance imaging of the pelvis (which covered the area between the diaphragm and the proximal femoral region), chest X‐ray or CT, abdomen ultrasonography, and a standard radionuclide bone scan. Positron emission tomography/CT was optional.

### 2.2 Surgical procedure

All patients had undergone a radical proctectomy with TME before adjuvant therapy. The choice between LAR and abdominoperineal resection (APR) was at the surgeons' discretion. Patients were informed about their treatment options, and, in some cases, the decision to undergo adjuvant RT was left to them. Two treatment pathways were created: an adjuvant CCRT arm and an adjuvant CTx arm.

### 2.3 Radiation therapy and chemotherapy

The RT procedure followed established protocols from previous studies.^23^ Briefly, the main component of CTx was fluorouracil, administered intravenously or orally. Combination regimens like FOLFOX (folinic acid, fluorouracil, and oxaliplatin) and FOLFIRI (folinic acid, fluorouracil, and irinotecan) were used. Generally, stage III patients were treated with FOLFOX, while stage II patients were given a fluorouracil + leucovorin or oral tegafur‐uracil + folinic acid regimen. Oxaliplatin and irinotecan were occasionally used. All patients who underwent irradiation received CCRT. Adjuvant CTx was sometimes administered before CCRT in patients with a high risk of distant metastasis.

### 2.4 Follow‐up

Post‐treatment, patients were followed up at 3‐month intervals. Radiological studies and fiberoptic colonoscopy were also performed every 3–6 months or in longer interval depending on clinical indications. Signs of possible recurrence were investigated through tumor markers. Recurrent lesions close to the anastomosis site were classified as local recurrence, and other lesions within the RT planned target volume were classified as regional recurrence (RR).

### 2.5 Statistical analysis

Collected demographic and clinical variables included sex, age, and tumor location. Histological variables comprised perineural invasion (PNI); lymphovascular invasion (LVI); surgical margins, including the CRM; and nodal invasion and retrieval status. Medical records were also examined for treatment complications, with the second cancer induction during follow‐up considered a related side effect. Side effects were categorized using the Common Terminology Criteria for Adverse Events (CTCAE) (v.5.0). Tumor deposit data, post the publication of the eighth edition of the American Joint Committee on Cancer staging manual in 2017, were available. A comprehensive list of variables measured is provided in Table 1.

**TABLE 1:** Clinical and demographic characteristics of the patients in our cohort with rectal cancer.

		CTx only (*n* = 135)	%	CCRT (*n* = 101)	%	*p* Value
Sex	Female	82	60.7	68	67.3	0.37
	Male	53	39.3	33	32.7	
Age		66.0 ± 11.3		60.4 ± 13.0		<0.001
Location	Proximal	76	56.3	33	32.7	0.001
	Distal	59	43.7	68	67.3	
Resection	LAR	133	98.5	97	96.0	0.44
	APR	2	1.5	4	4.0	
Differentiation	Well	2	1.48	1	1.0	0.112
	Moderate	130	96.3	91	91.0	
	Poor	3	2.22	8	8.0	
LVI	No	34	25.2	15	14.9	0.099
	Yes	100	74.1	86	85.2	
PNI	No	86	63.8	56	55.5	0.64
	Yes	41	30.4	38	37.6	
CEA^a^		9.8 ± 38.6		7.7 ± 14.7		0.615
	<5	89	70.6	62	63.3	0.306
	≥5	37	39.4	36	36.7	
Size in mm^a^		49.9 ± 18.9		46.0 ± 19.1		0.119
pN stage	N0	65	48.2	28	27.7	<0.001
	N1	48	35.6	33	32.7	
	N2	22	16.3	40	39.6	
Perforation	No	131	97.0	96	95.1	0.656
	Yes	4	3.0	5	5.0	
Obstruction	No	101	74.8	66	65.4	0.151
	Yes	34	25.2	35	34.7	
Comorbidity	No	40	29.9	33	33.0	0.71
	Yes	94	70.2	67	67.0	
ECOG score	<2	118	87.4	89	88.1	1
	≥2	17	12.6	12	11.9	

Abbreviations: APR: abdominoperineal resection; LAR: low anterior resection; LVI: lymphovascular invasion; PNI: perineural invasion; LN: lymph node; CTx: chemotherapy; CCRT: concurrent chemoradiation; IV: intravenous; CEA: initial serum carcinoembryonic antigen level; pN stage: pathological nodal stage; ECOG: initial Eastern Cooperative Oncology Group Performance Status score.

^a^ mean ± standard deviation.

Primary endpoints were DFS, with secondary endpoints being OS, local recurrence‐free survival (LRFS), regional recurrence‐free survival (RRFS), LRRFS, and distant metastasis‐free survival (DMFS). For the CCRT arm, survival duration was calculated from the end of RT until either death or the last follow‐up. The Kaplan–Meier method was used for creating survival curves, and the differences between the curves were compared using log‐rank tests. A Cox proportional hazard regression model was used to calculate hazard ratios and the corresponding 95% confidence interval. If the survival curves on a Kaplan–Meier plot intersect, we verify it using Schoenfeld residuals to test the assumption of proportional hazards. Chi‐square or independent *t*‐tests (or Fisher's exact test for small cell sizes) were used to compare differences between groups for categorical and continuous variables. All tests were two‐sided, with *p* values <0.05 deemed significant. All statistical analyses were conducted using R software, version 4.1.2 (R Foundation for Statistical Computing, Vienna, Austria).

Following the analysis of the two arms of the whole cohort, a subgroup survival analysis was performed for proximal and distal (middle and low) rectal cancer patients. Tumors located below the level of peritoneal reflection or 10 cm AAV were defined as distal (which included middle and low region, defined as >5 cm and ≤5 cm AAV, respectively) rectal cancer; all others were classified as proximal.^24^

## 3 RESULTS

### 3.1 Patient demographics and clinical features

Table 1 presents the demographic and clinical traits of the two patient groups diagnosed with rectal cancer. Our study included 236 rectal adenocarcinoma patients (101 receiving CCRT and 135 receiving CTx). A significant likelihood of not undergoing RT was observed in patients with fewer involved lymph nodes, older age (*p* < 0.001), and proximal tumors location (*p* = 0.001). However, no significant discrepancies were found in other variables between the two groups. The median duration of follow‐up was 4.9 years, with an interquartile range of 2.4–7.5 years. Among these, 88 were diagnosed with pT3N0 rectal cancer (26 in the CCRT group and 62 in the CTx group which included 37 proximal‐located cases), with the details shown in Table S1.

### 3.2 Effectiveness of treatments

**FIGURE 1:**
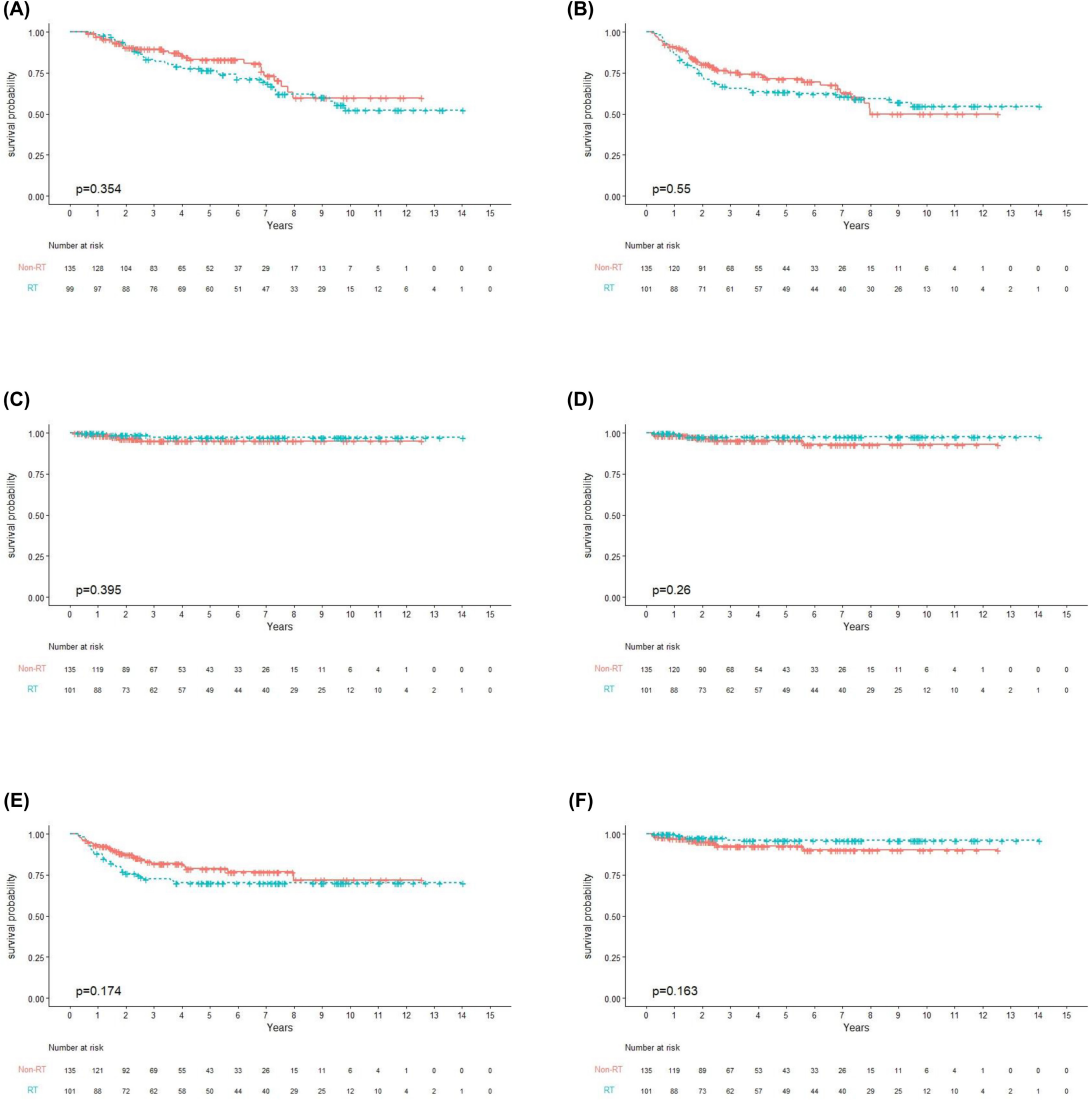
Kaplan–Meier plots, including the number at risk, depict the outcomes of 236 patients with stage II and III rectal adenocarcinoma (CCRT: 101; CTx: 135) who underwent postoperative treatment with either chemotherapy alone or concurrent chemoradiation (A) Overall survival; (B) Disease progression‐free survival; (C) Local relapse‐free survival; (D) Regional relapse‐free survival; (E) Distant metastasis‐free survival; (F) Locoregional relapse‐free survival. RT, radiotherapy; CCRT, concurrent chemoradiation; CTx, chemotherapy.

No statistically significant differences were identified in any of the survival outcomes (OS, DFS, LRFS, RRFS, LRRFS, and DMFS) between the treatment groups, either for the whole group (Figure 1A–F) comparisons. The same trends were observed when comparing distal and proximal subgroups, and risk‐stratified subgroups (Figures S1–S14A–F). Table S2 includes details of the 12 cases of recurrence, which comprised both local and regional cases. The 10‐year LRRFS for the two arms of the entire cohort were 90.1% for the CTx arm and 96.1% for the CCRT arm, with no statistically significant difference (*p* = 0.163; Figure 1F). However, after performing multivariate adjustments, the CCRT group exhibited a nonsignificant tendency toward better locoregional control after multivariate adjustments (*p* = 0.052; Table 2).

**TABLE 2:** Univariate and multivariate Cox regression analyses of locoregional relapse‐free survival in a cohort of rectal adenocarcinoma patients.

	Crude HR (95% CI)	*p*	Adjusted HR (95% CI)	*p*
CCRT (reference: CTx alone)	0.41 (0.11–1.50)	0.177	0.18 (0.03–1.02)	0.052
Male (reference: female)	0.83 (0.25–2.75)	0.755	0.44 (0.10–1.92)	0.274
Age	1.05 (0.99–1.10)	0.094	1.08 (1.00–1.16)	0.041
Distal rectum (reference: proximal)	2.66 (0.72–9.82)	0.142	5.53 (0.96–31.85)	0.055
APR (reference: LAR)	3.63 (0.47–28.20)	0.217	0.30 (0.00–28.66)	0.606
Poorly differentiated	2.59 (0.78–8.59)	0.120	0.83 (0.08–8.13)	0.871
Lymphovascular invasion (reference: no)	1.41 (0.31–6.44)	0.657	1.73 (0.19–15.58)	0.624
Perineural invasion (reference: no)	1.74 (0.56–5.39)	0.339	1.67 (0.35–8.08)	0.523
CEA ≥5 (reference: <5)	1.20 (0.36–4.00)	0.763	1.42 (0.26–7.92)	0.686
Maximal size	1.00 (0.97–1.04)	0.856	0.98 (0.94–1.02)	0.379
Perforation (reference: no)	12.20 (3.28–45.50)	< 0.001	72.80 (3.04–1746.25)	0.008
Obstruction (reference: no)	0.53 (0.12–2.43)	0.415	0.87 (0.14–5.36)	0.880
ECOG score ≥2 (reference: <2)	1.48 (0.32–6.76)	0.613	1.11 (0.12–10.67)	0.925
Lymph node involvement ratio	11.70 (1.69–81.30)	0.013	3.90 (0.16–96.30)	0.405
T3, 4 (reference: T1, 2)	0.70 (0.15–3.22)	0.651	0.35 (0.05–2.34)	0.279

Abbreviations: APR, abdominoperineal resection; CCRT, concurrent chemoradiation; CEA, initial serum carcinoembryonic antigen level; CI, confidence interval; CRM, circumferential resection margin; CTx, chemotherapy; ECOG score, initial Eastern Cooperative Oncology Group Performance Status score; HR, hazard ratio; IV, intravenous; LAR, low anterior resection; LN, lymph node.

Table 3 provide the frequency, location of the first recurrence, and of initial salvage treatment modality for local and regional recurrences and distant failures, respectively, for an entire cohort of patients with rectal cancer. Of total 236 patients, 54 (22.9%) had distant lesions, primarily located in the lung, liver, and bone. More specifically, 13 cases were from the proximal rectum and 12 from distal region in the CTx arm, whereas in the CCRT arm, nine cases were from the proximal region and 20 from distal region (Tables S3 and S4).

**TABLE 3:** Locoregional and distant relapses in whole cohort patients. Recurrence frequency, site of the first recurrence, and the first salvage treatment modality are shown.

	Adjuvant CTx only		Adjuvant CCRT	
	*n* = 135		*n* = 101	
	No.	%	No.	%
First failure site				
Local	3	2.2	1	1.0
Regional	3	2.2	1	1.0
Local + regional	2	1.5	0	0
Locoregional + distant	1	0.7	1	1.0
Distant	24	17.0	28	27.7
First treatment modality for locoregional relapse				
OP + CTx	1	11	0	0
RT+ CTx	1	11	0	0
CTx	2	22	1	33
OP	1	11	0	0
OP + RT	1	11	0	0
Nil	3	33	2	67

Abbreviations: CCRT: concurrent chemoradiation; CTx: chemotherapy; OP: operation; RT: radiotherapy.

In the 44 proximal pT3N0 rectal cancer patients (37 undergoing CTx and seven receiving CCRT), RR was observed in one CTx patient, diagnosed with sigmoid colon cancer located 40 cm from the anal verge. Among the 44 distal pT3N0 patients (25 CTx, 19 CCRT), two CTx patients experienced local recurrence, with one having simultaneous RR. For all 88 pT3N0 patients, no cases of isolated RR were reported in the CCRT group. (Figures S12–S14A–F).

### 3.3 Treatment side effects

Patients in the CCRT group exhibited significantly more grade ≥3 side effects, both acute and chronic (*p* < 0.001 and = 0.009, respectively; Table 4). Additionally, this trend was observed in the 88 pT3N0 patients, particularly for complications like diarrhea (*p* = 0.041; Table S5). Second cancers were reported in nine cases (five in the CTx arm and four in the CCRT arm). However, more cancer‐related deaths occurred in the CCRT arm (two deaths, 2%) than in the CTx arm (one death, 1%).

**TABLE 4:** Side effects in a cohort of rectal adenocarcinoma patients treated with chemotherapy alone or chemotherapy and intensity‐modulated radiotherapy after total mesorectum excision. Side effects were classified using the Common Terminology Criteria for Adverse Events (CTCAE) (v.5.0).

	CTx alone	CCRT	*p*
	*n* = 135 (%)	*n* = 101 (%)	
Acute			
Acute G3 diarrhea	8 (5.9)	30 (29.7)	<0.001
Acute G3 Hematologic	10 (7.4)	10 (9.9)	0.657
Acute G3 skin	1 (0.7)	7 (6.9)	0.025
Acute G3 any	40 (29.6)	48 (47.5)	0.007
Long‐term			
Long‐term G3 GI	5 (3.7)	11 (10.9)	0.056
Long‐term G3 stricture Anastomosis	2 (1.48)	3 (3.0)	0.742
Long‐term G3 bladder	0 (0.00)	1 (0.99)	0.884
Long‐term G3 any	12 (8.9)	22 (21.8)	0.009
2nd cancer grade			
3	1 (0.75)	2 (1.94)	0.823
4	3 (2.2)	0 (0.0)	0.357
5	1 (0.7)	2 (2.0)	0.800

Abbreviations: CCRT, concurrent chemoradiation; CTx, chemotherapy; G3, grade 3.

### 3.4 Prognostic factors

Both univariate and multivariate Cox regression analyses were performed using data from the entire cohort. The significant risk factors for both OS and DFS, older age, the ratio of positive LNs, and perforation were consistently significant. Tumor size and the ratio of positive LNs were found to be consistently statistically significant factors affecting DMFS (Tables S6–S8). In contrast only perforation were consistently significant factor, whereas multivariate analyses the absence of RT (*p* = 0.052) exhibited a trend concerning LRRFS (Table 2). However, two factors, namely upper tumor location and LVI, exhibited significant violations of the proportional hazard assumption (Table S9). Consequently, we attempted stratification of these two factors. The subsequent separate Cox test results revealed no RT (*p* = 0.038), older age (*p* = 0.012), and perforation (*p* = 0.001) to be significant factors for LRRFS in the multivariate Cox regression analyses (Table S10). No other parameters, including sex, T stage, comorbidities, serum carcinoembryonic antigen levels, or ECOG performance scores, showed a consistently significant relationship with outcomes.

## 4 DISCUSSION

In our cohort of patients who had undergone comprehensive TMEs, we identified similar oncological results in those treated postoperatively with CTx and CCRT, including the pT3N0 upper rectal cancer subgroup (Figures 1A–F). Among the 44 upper rectal pT3N0 cases, we observed nearly equivalent survival outcomes, especially in LRFS, RRFS, and LRRFS (Figures S13A–F).

The risk of locoregional recurrence (LRR) due to complete TME was less than 10%, indicated by the LRRFS for the CTx group. Hence, further improvement with postoperative treatment is challenging.^25^, ^26^ The only instance of LRR among the 37 cases of upper rectal pT3N0 patients who received CTx was likely a diathesis and would not have been preventable by adding RT to CTx. Older patients were less likely to choose CCRT, and our data showed advanced age significantly linked with lower OS, which could have led to an overestimation of OS in the CCRT arm.

The CCRT arm displayed a higher incidence of severe, long‐term complications and acute side effects. The RT‐induced Malignant Mixed Müllerian Tumor (MMMT) caused a fatality 7 years after RT was performed,^27^ showing that using RT for locoregional control carries long‐term survival risks. The cancer induction risk from RT complications seen in the cohort can be reasonably extended to the pT3N0 group, as their RT volumes and doses were virtually identical. Despite the limited number of cases, based on our findings and NCCN guidelines, we suggest that CTx alone is the more appropriate treatment option for upper rectal pT3N0 cancer following adequate TME.

Various studies have identified risk factors for LRR in pT3N0 upper rectal cancer patients.^10^, ^11^, ^12^, ^13^, ^14^, ^17^, ^28^, ^29^, ^30^, ^31^ However, perforation was the most significant risk factor (Table 2 and Table S10). Perforation itself can directly disrupt the mesorectal fascial envelope, challenging locoregional control. Later, two out of eight perforation cases in our cohort (25%) developed LRR, even though five of them had received adjuvant CCRT. This rate is consistent with surgical outcomes before the era of TME.^6^, ^7^ Although perforation does not necessarily indicate a tumor beyond pT3N0, it negatively impacts tumor control and should be considered when deciding on RT treatment in these patients.^10^, ^28^ Additionally, in our results, older age was significant for LRR (Table 2 and Table S10). Nevertheless, we could not find any other strong reasons to determine whether or not to pursue RT. Therefore, further investigations are warranted.

Although our study and previous ones have relatively small sample sizes, assessing recurrence risk in upper rectal pT3N0 patients should consider the variables correlated with recurrence unless other evidence argues against using RT in a particular case. While adjuvant RT does not seem to provide additional benefits over CTx alone, it could be appropriate for pT3N0 upper rectal cancer patients with perforated tumors or an extremely high risk of LRR. In cases where RT is necessary, minimizing the toxicity of postoperative CCRT is essential.

This study explored the potential benefits of postoperative CCRT by comparing it with CTx alone in different risk strata, including patients with proximal or distal rectal cancer. Despite the limitations of this study, which include a limited number of cases and potential selection bias due to the nonrandomized study design, our analyses found no evidence to support concurrent treatment of rectal adenocarcinoma with RT alongside postoperative CTx.

There was at least a noticeable trend toward better LRRFS in the CCRT arm of the study cohort, mildly supporting the current NCCN recommendation of adjuvant CCRT for patients with distal pT3 or pT4 or pN+ cancers. Future investigations should confirm our results.

Given the continued evolution of perioperative RT and recent notable results using upfront surgery,^1^, ^5^ further research on the use of postoperative CCRT after upfront TME in larger patient cohorts and subgroup analyses based on tumor locations and risk stratification of patients with LARC is warranted.

## 5 CONCLUSION

Our research indicates that patients with upper rectal cancer categorized as pT3N0, who have undergone a thorough TME and received adjuvant CTx, may not necessitate the application of postoperative IMRT. This conclusion stems from the comparable oncological outcomes observed in patients treated with CTx alone or combined with RT in our study cohort. It is particularly true without specific risk factors, such as perforation or an extremely high likelihood of LRR. Our study emphasizes the importance of further research to validate these findings and to establish more definitive guidelines for the postoperative management of pT3N0 upper rectal cancer patients.

## AUTHOR CONTRIBUTIONS

**Chuan‐Yin Fang:** Conceptualization (equal); writing – original draft (equal). **Hsuan‐yu Chen:** Formal analysis (equal); methodology (equal). **Hsin‐Yi Yang:** Formal analysis (equal); investigation (equal); validation (equal). **Yuk‐Wah Tsang:** Data curation (equal); investigation (equal). **Cheng‐Yen Lee:** Data curation (equal); investigation (equal). **Chih‐Chia Chang:** Data curation (equal); investigation (equal). **I‐Chen Lin:** Data curation (equal); validation (equal). **Yun‐Jhong Huang:** Data curation (equal). **Chun‐Ting Chu:** Data curation (equal); supervision (equal); visualization (equal); writing – review and editing (equal). **Yu‐Wen Wang:** Conceptualization (equal); data curation (equal); funding acquisition (equal); project administration (equal); supervision (equal); writing – original draft (equal); writing – review and editing (equal).

## FUNDING INFORMATION

This work was supported by a grant from the Ditmanson Medical Foundation Chia‐Yi Christian Hospital Research Program [grant number: R111‐005 to YWW].

## CONFLICT OF INTEREST STATEMENT

All authors declare having no conflict of interest related to the content of this manuscript.

## ETHICS STATEMENT

This work has been carried out following the Code of Ethics of the World Medical Association (Declaration of Helsinki) for experiments involving humans. Based on the review of medical records, this study has been granted an exemption from the requirement of written informed consent and has been approved by the Institutional Review Board at Ditmanson Medical Foundation Chia‐Yi Christian Hospital (CYCH‐IRB 2020147).

## Supporting information


Figures S1–S14

Tables S1–S10

## Data Availability

The data that support the findings of this study are available on request from the corresponding authors, [YWW] or [CTC], upon reasonable request.

## References

[1] Network NCC . Rectal Cancer NCCN Guidelines, 2023; Version 3. 2023 Available online: https://www.nccn.org/professionals/physician_gls/PDF/rectal.pdf

[2] Roh MS , Colangelo LH , O'Connell MJ , et al. Preoperative multimodality therapy improves disease‐free survival in patients with carcinoma of the rectum: NSABP R‐03. J Clin Oncol. 2009;27(31):5124‐5130. 19770376 10.1200/JCO.2009.22.0467 PMC2773471

[3] Park JH , Yoon SM , Yu CS , Kim JH , Kim TW , Kim JC . Randomized phase 3 trial comparing preoperative and postoperative chemoradiotherapy with capecitabine for locally advanced rectal cancer. Cancer. 2011;117(16):3703‐3712. 21328328 10.1002/cncr.25943

[4] Sauer R , Liersch T , Merkel S , et al. Preoperative versus postoperative chemoradiotherapy for locally advanced rectal cancer: results of the German CAO/ARO/AIO‐94 randomized phase III trial after a median follow‐up of 11 years. J Clin Oncol. 2012;30(16):1926‐1933. 22529255 10.1200/JCO.2011.40.1836

[5] Li N , Zhu Y , Liu LY , et al. Postoperative Chemoradiotherapy with Capecitabine and Oxaliplatin vs Capecitabine for stage II to III rectal cancer: a randomized clinical trial. JAMA Netw Open. 2021;4(11):e2136116. 34846525 10.1001/jamanetworkopen.2021.36116 PMC8634060

[6] Thomas PR , Lindblad AS . Adjuvant postoperative radiotherapy and chemotherapy in rectal carcinoma: a review of the gastrointestinal tumor study group experience. Radiother Oncol. 1988;13(4):245‐252. 3064191 10.1016/0167-8140(88)90219-8

[7] Group CCC . Adjuvant radiotherapy for rectal cancer: a systematic overview of 8,507 patients from 22 randomised trials. Lancet (London, England). 2001;358(9290):1291‐1304. 11684209 10.1016/S0140-6736(01)06409-1

[8] Cedermark B , Johansson H , Rutqvist LE , Wilking N . The Stockholm I trial of preoperative short term radiotherapy in operable rectal carcinoma. A prospective randomized trial. Stockholm colorectal cancer study group. Cancer. 1995;75(9):2269‐2275. 7712435 10.1002/1097-0142(19950501)75:9<2269::aid-cncr2820750913>3.0.co;2-i

[9] Tepper JE , O'Connell M , Niedzwiecki D , et al. Adjuvant therapy in rectal cancer: analysis of stage, sex, and local control—final report of intergroup 0114. J Clin Oncol. 2002;20(7):1744‐1750. 11919230 10.1200/JCO.2002.07.132

[10] Lai LL , Fuller CD , Kachnic LA , Thomas CR Jr . Can pelvic radiotherapy be omitted in select patients with rectal cancer? Semin Oncol. 2006;33(6 Suppl 11):S70‐S74. 17178292 10.1053/j.seminoncol.2006.10.019

[11] Bokey EL , Ojerskog B , Chapuis PH , Dent OF , Newland RC , Sinclair G . Local recurrence after curative excision of the rectum for cancer without adjuvant therapy: role of total anatomical dissection. Br J Surg. 1999;86(9):1164‐1170. 10504371 10.1046/j.1365-2168.1999.01216.x

[12] Merchant NB , Guillem JG , Paty PB , et al. T3N0 rectal cancer: results following sharp mesorectal excision and no adjuvant therapy. J Gastrointest Surg. 1999;3(6):642‐647. 10554372 10.1016/s1091-255x(99)80087-0

[13] Nissan A , Stojadinovic A , Shia J , et al. Predictors of recurrence in patients with T2 and early T3, N0 adenocarcinoma of the rectum treated by surgery alone. J Clin Oncol. 2006;24(25):4078‐4084. 16943525 10.1200/JCO.2006.06.2968

[14] Peng HH , Zhou XH , Zhou TC , Qiu XS , You KY . Tumor location as an indication for adjuvant radiotherapy in pT3N0 rectal cancer after surgery. Radiat Oncol. 2019;14(1):8. 30651116 10.1186/s13014-019-1206-3 PMC6334427

[15] Fisher B , Wolmark N , Rockette H , et al. Postoperative adjuvant chemotherapy or radiation therapy for rectal cancer: results from NSABP protocol R‐01. J Natl Cancer Inst. 1988;80(1):21‐29. 3276900 10.1093/jnci/80.1.21

[16] Gunderson LL , Sargent DJ , Tepper JE , et al. Impact of T and N stage and treatment on survival and relapse in adjuvant rectal cancer: a pooled analysis. J Clin Oncol. 2004;22(10):1785‐1796. 15067027 10.1200/JCO.2004.08.173

[17] Park IJ , Kim HC , Yu CS , Kim TW , Jang SJ , Kim JC . Effect of adjuvant radiotherapy on local recurrence in stage II rectal cancer. Ann Surg Oncol. 2008;15(2):519‐525. 17960464 10.1245/s10434-007-9643-x

[18] Faerden AE , Naimy N , Wiik P , et al. Total mesorectal excision for rectal cancer: difference in outcome for low and high rectal cancer. Dis Colon Rectum. 2005;48(12):2224‐2231. 16228823 10.1007/s10350-005-0191-9

[19] Oronsky B , Reid T , Larson C , Knox SJ . Locally advanced rectal cancer: the past, present, and future. Semin Oncol. 2020;47(1):85‐92. 32147127 10.1053/j.seminoncol.2020.02.001

[20] Kim JS , Kim NK , Min BS , Hur H , Ahn JB , Keum KC . Adjuvant radiotherapy following total mesorectal excision for stage IIA rectal cancer: is it beneficial? Int J Color Dis. 2010;25(9):1103‐1110. 10.1007/s00384-010-0970-1 20544208

[21] Huang YX , Lin YZ , Li JL , et al. Role of postoperative radiotherapy in pT3N0 rectal cancer: a risk‐stratification system based on population analyses. Cancer Med. 2019;8(3):1024‐1033. 30714683 10.1002/cam4.1991 PMC6434337

[22] Quinn TJ , Rajagopalan MS , Gill B , Mehdiabadi SM , Kabolizadeh P . Patterns of care and outcomes for adjuvant treatment of pT3N0 rectal cancer using the National Cancer Database. J Gastrointest Oncol. 2020;11(1):1‐12. 32175100 10.21037/jgo.2019.10.02 PMC7052766

[23] Lee CY , Chang CC , Yang HY , Chiang PY , Tsang YW . Intensity modulated radiotherapy delivers competitive local control rate with limited acute toxicity in the adjuvant treatment of rectal cancer. Jpn J Clin Oncol. 2018;48(7):653‐660. 29868768 10.1093/jjco/hyy075

[24] Yiqun S , Tong T , Fangqi L , et al. Recognition of anterior peritoneal reflections and their relationship with rectal tumors using rectal magnetic resonance imaging. Medicine. 2016;95(9):e2889. 26945377 10.1097/MD.0000000000002889 PMC4782861

[25] Sauer R . Adjuvant and neoadjuvant radiotherapy and concurrent radiochemotherapy for rectal cancer. Pathol Oncol Res. 2002;8(1):7‐17. 11994757 10.1007/BF03033695

[26] Vonk DT , Hazard LJ . Do all locally advanced rectal cancers require radiation? A review of literature in the modern era. J Gastrointest Oncol. 2010;1(1):45‐54. 22811804 10.3978/j.issn.2078-6891.2010.008 PMC3397573

[27] Huang Y‐T , Huang K‐G , Ueng S‐H , Shaw S‐W . Irradiation‐induced uterine malignant mixed Müllerian tumor. Taiwan J Obstet Gynecol. 2006;45(4):353‐355. 17175499 10.1016/S1028-4559(09)60260-6

[28] Jörgren F , Lydrup ML , Buchwald P . Impact of rectal perforation on recurrence during rectal cancer surgery in a national population registry. Br J Surg. 2020;107(13):1818‐1825. 32484249 10.1002/bjs.11710

[29] Willett CG , Badizadegan K , Ancukiewicz M , Shellito PC . Prognostic factors in stage T3N0 rectal cancer: do all patients require postoperative pelvic irradiation and chemotherapy? Dis Colon Rectum. 1999;42(2):167‐173. 10211491 10.1007/BF02237122

[30] Horn A , Dahl O , Morild I . Venous and neural invasion as predictors of recurrence in rectal adenocarcinoma. Dis Colon Rectum. 1991;34(9):798‐804. 1914747 10.1007/BF02051074

[31] Enker WE , Thaler HT , Cranor ML , Polyak T . Total mesorectal excision in the operative treatment of carcinoma of the rectum. J Am Coll Surg. 1995;181(4):335‐346. 7551328

