# Supplementary material for: The impact of intensity‐modulated radiotherapy in conjunction with chemotherapy on proximal pT3N0 rectal cancer patients after total mesorectum excision
Source: Cancer Med. 2023 Nov 6;12(23):21209–18. doi: 10.1002/cam4.6691 (PMC10726884; doi:10.1002/cam4.6691)
Supplement: Supplementary file 1 — Figures S1–S14 [file CAM4-12-21209-s002.docx]

**SUPPLEMENT FIGURES:**


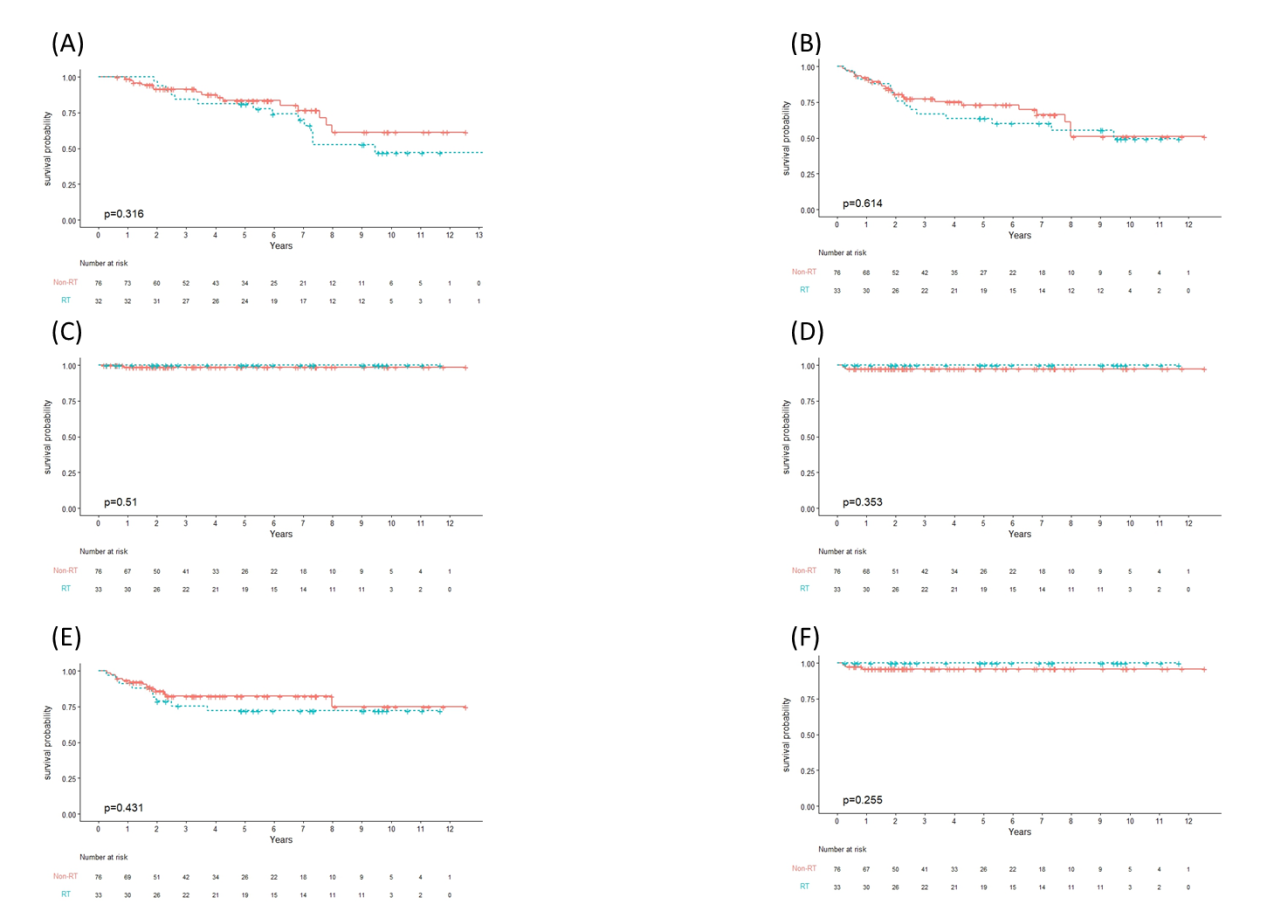


**FIGURE S1.** Kaplan-Meier Plots showing the results of 109(CCRT: 33; CTx: 76) proximal rectal adenocarcinoma patients (**A**) Overall survival; (**B**) Disease progression-free survival; (**C**) Local relapse-free survival; (**D**) Regional relapse-free survival; (**E**) Distant metastasis-free survival; (**F**) Locoregional relapse-free survival. RT, radiotherapy; CCRT, concurrent chemoradiation; CTx, chemotherapy.


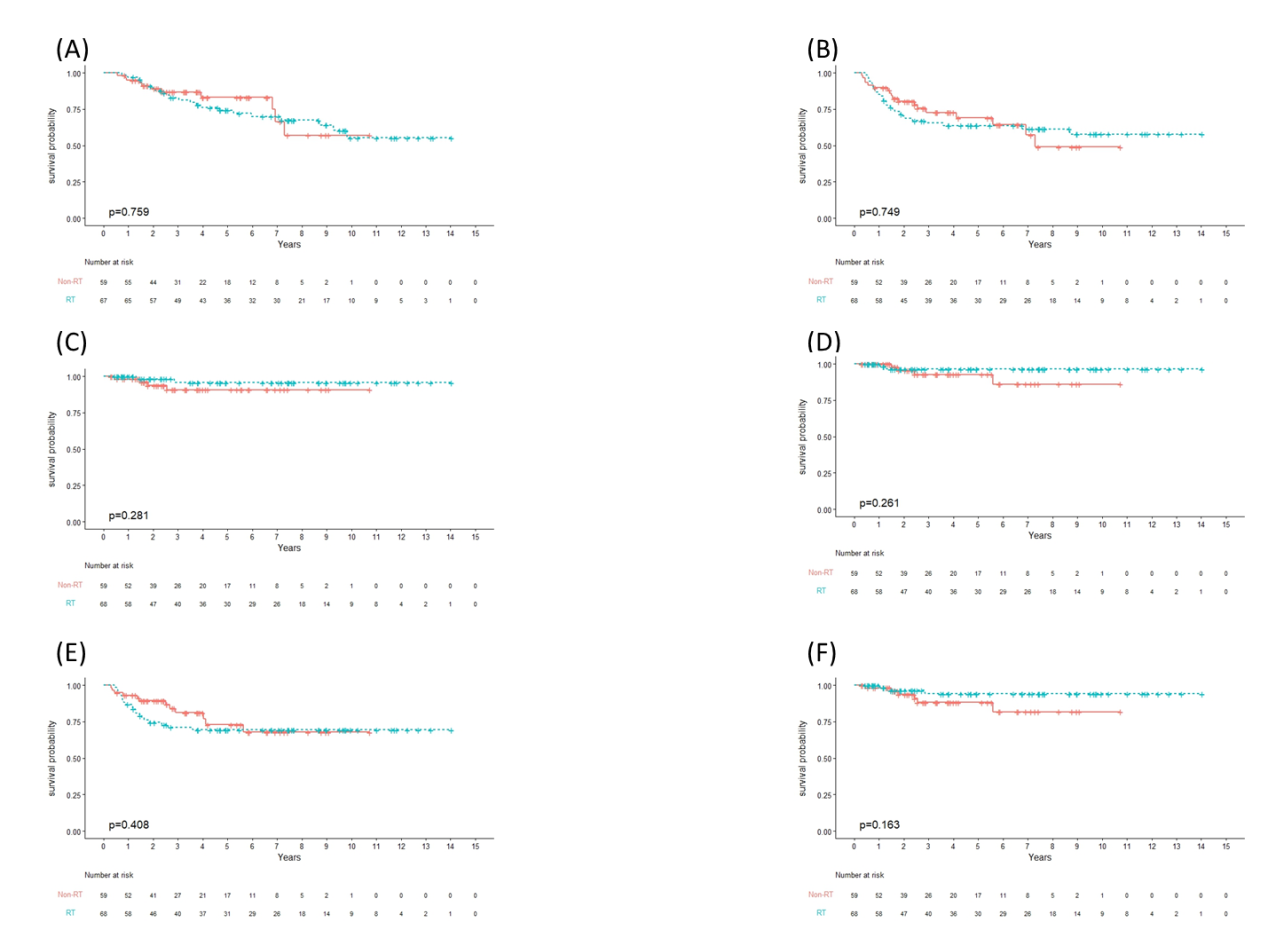


**FIGURE S2.** Kaplan-Meier Plots showing the results of 127 (CCRT: 68; CTx: 59) middle and low rectal adenocarcinoma patients (**A**) Overall survival; (**B**) Disease progression-free survival; (**C**) Local relapse-free survival; (**d**) Regional relapse-free survival; (**E**) Distant metastasis-free survival; (**F**) Locoregional relapse-free survival. RT, radiotherapy; CCRT, concurrent chemoradiation; CTx, chemotherapy.


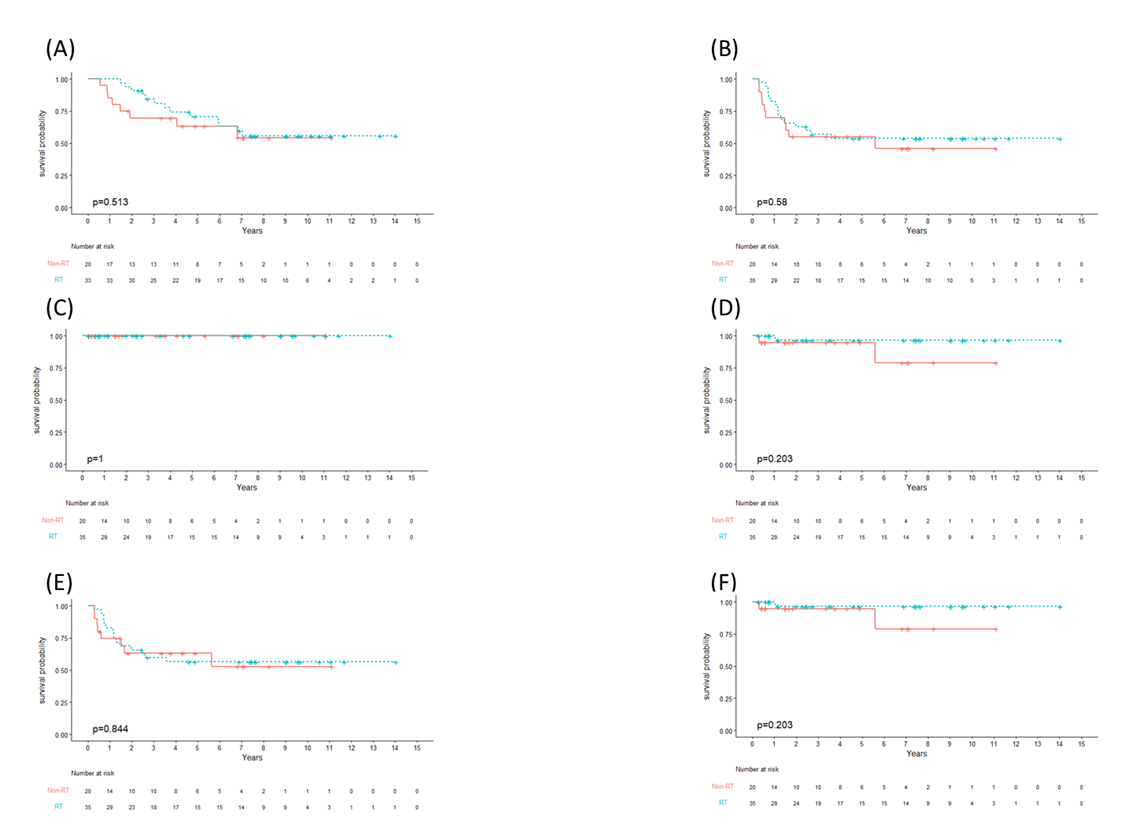


**FIGURE S3.** Kaplan-Meier Plots showing the results of 55 (CCRT: 35; CTx: 20) high risk (T3~4N2, T4N1) rectal adenocarcinoma patients (**A**) Overall survival; (**B**) Disease progression-free survival; (**C**) Local relapse-free survival; (**D**) Regional relapse-free survival; (**E**) Distant metastasis-free survival; (**F**) Locoregional relapse-free survival. RT, radiotherapy; CCRT, concurrent chemoradiation; CTx, chemotherapy.


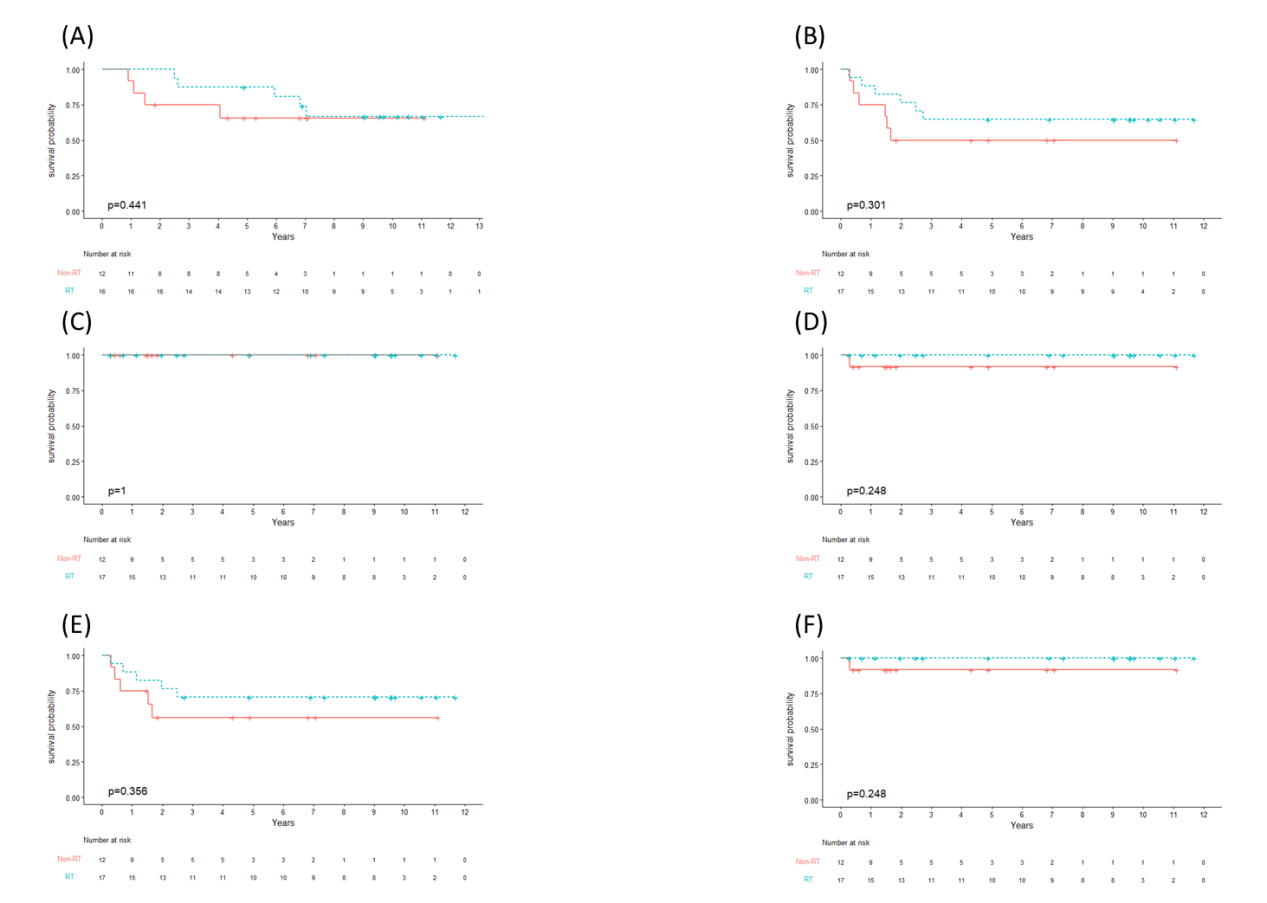


**FIGURE S4.** Kaplan-Meier Plots showing the results of 29 (CCRT: 17; CTx: 12) high risk (T3~4N2, T4N1) proximal rectal adenocarcinoma patients (**A**) Overall survival; (**B**) Disease progression-free survival; (**C**) Local relapse-free survival; (**D**) Regional relapse-free survival; (**E**) Distant metastasis-free survival; (**F**) Locoregional relapse-free survival. RT, radiotherapy; CCRT, concurrent chemoradiation; CTx, chemotherapy.


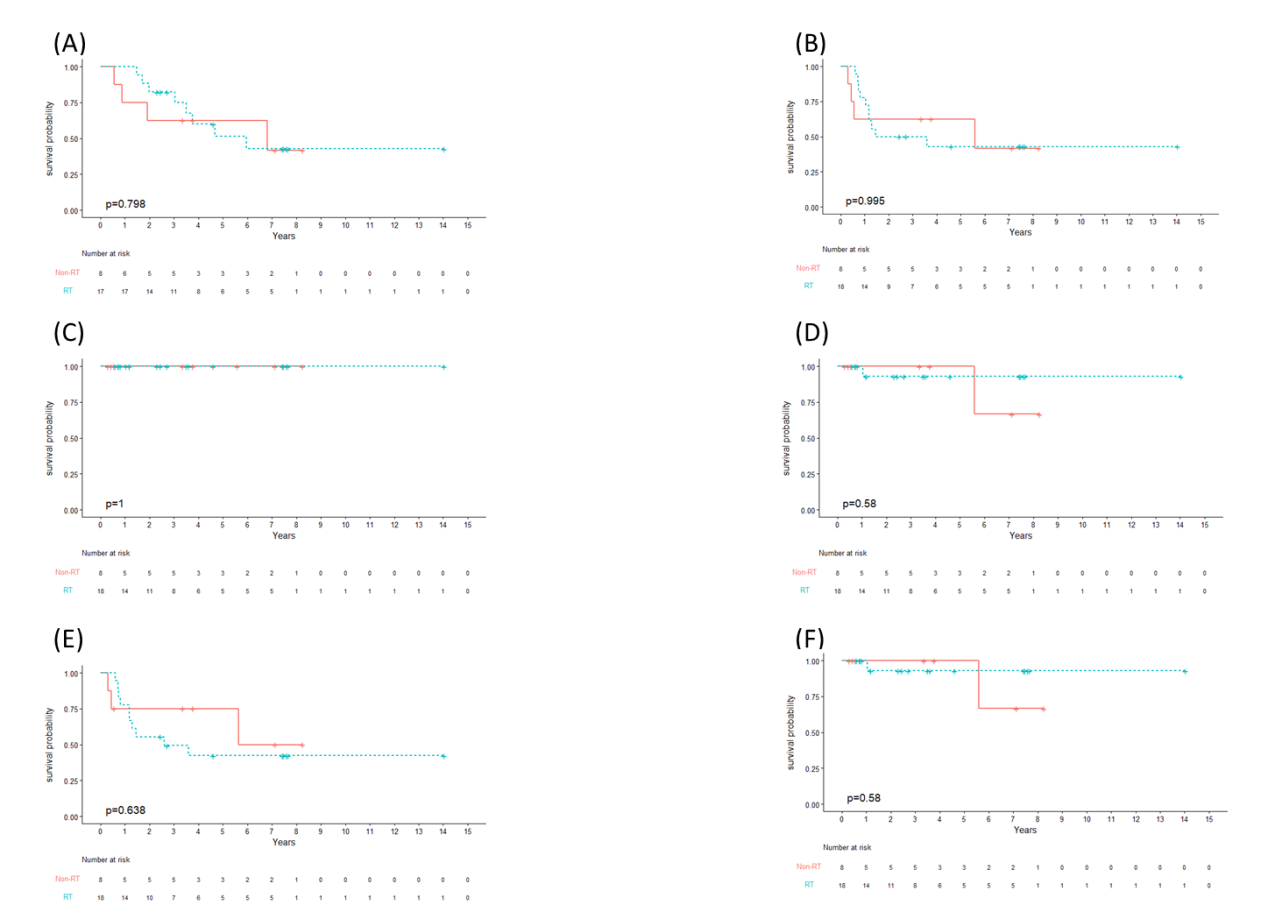


**FIGURE S5.** Kaplan-Meier Plots showing the results of 26 (CCRT: 18; CTx: 8) high risk (T3~4N2, T4N1) middle and low rectal adenocarcinoma patients (**A**) Overall survival; (**B**) Disease progression-free survival; (**C**) Local relapse-free survival; (**D**) Regional relapse-free survival; (**E**) Distant metastasis-free survival; (**F**) Locoregional relapse-free survival. RT, radiotherapy; CCRT, concurrent chemoradiation; CTx, chemotherapy.


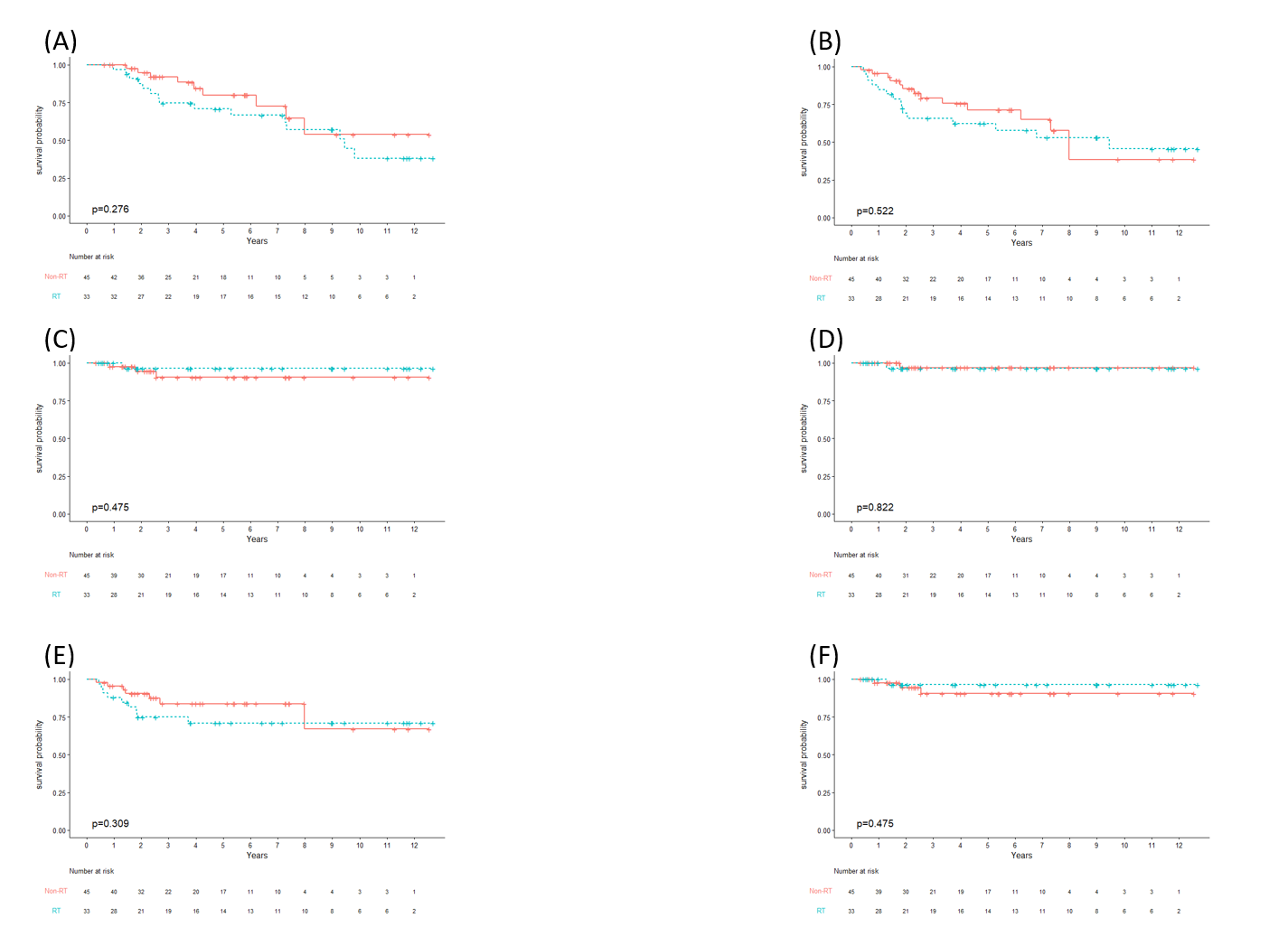


**FIGURE S6.** Kaplan-Meier Plots showing the results of 78 (CCRT: 33; CTx: 45) moderately high risk (T3N1, T4N0, T1N2, T2N2) rectal adenocarcinoma patients (**A**) Overall survival; (**B**) Disease progression-free survival; (**C**) Local relapse-free survival; (**D**) Regional relapse-free survival; (**E**) Distant metastasis-free survival; (**F**) Locoregional relapse-free survival. RT, radiotherapy; CCRT, concurrent chemoradiation; CTx, chemotherapy.


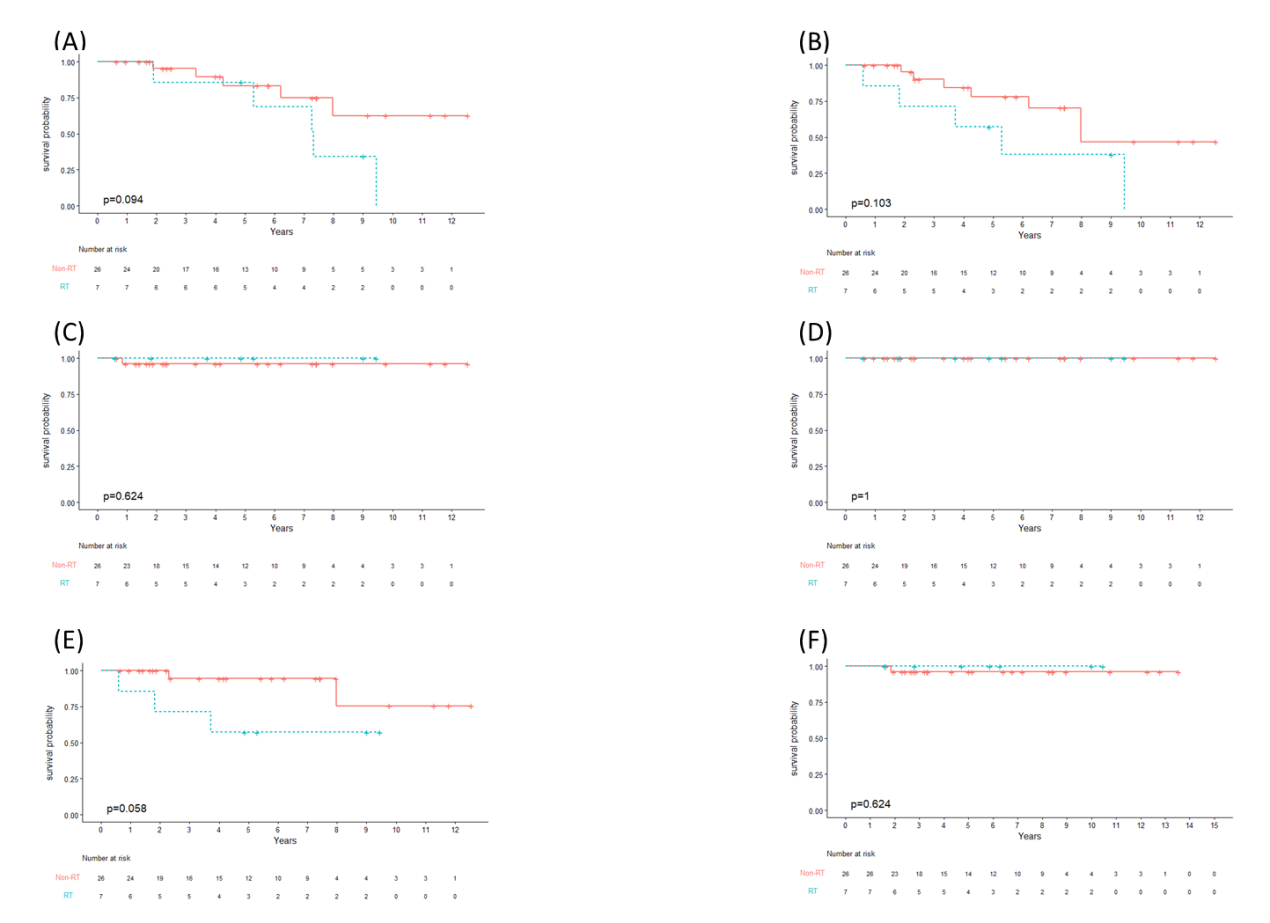


**FIGURE S7.** Kaplan-Meier Plots showing the results of 33 (CCRT: 7; CTx: 26) moderately high risk (T3N1, T4N0, T1N2, T2N2) proximal rectal adenocarcinoma patients (**A**) Overall survival; (**B**) Disease progression-free survival; (**C**) Local relapse-free survival; (**D**) Regional relapse-free survival; (E) Distant metastasis-free survival; (F) Locoregional relapse-free survival. RT, radiotherapy; CCRT, concurrent chemoradiation; CTx, chemotherapy.


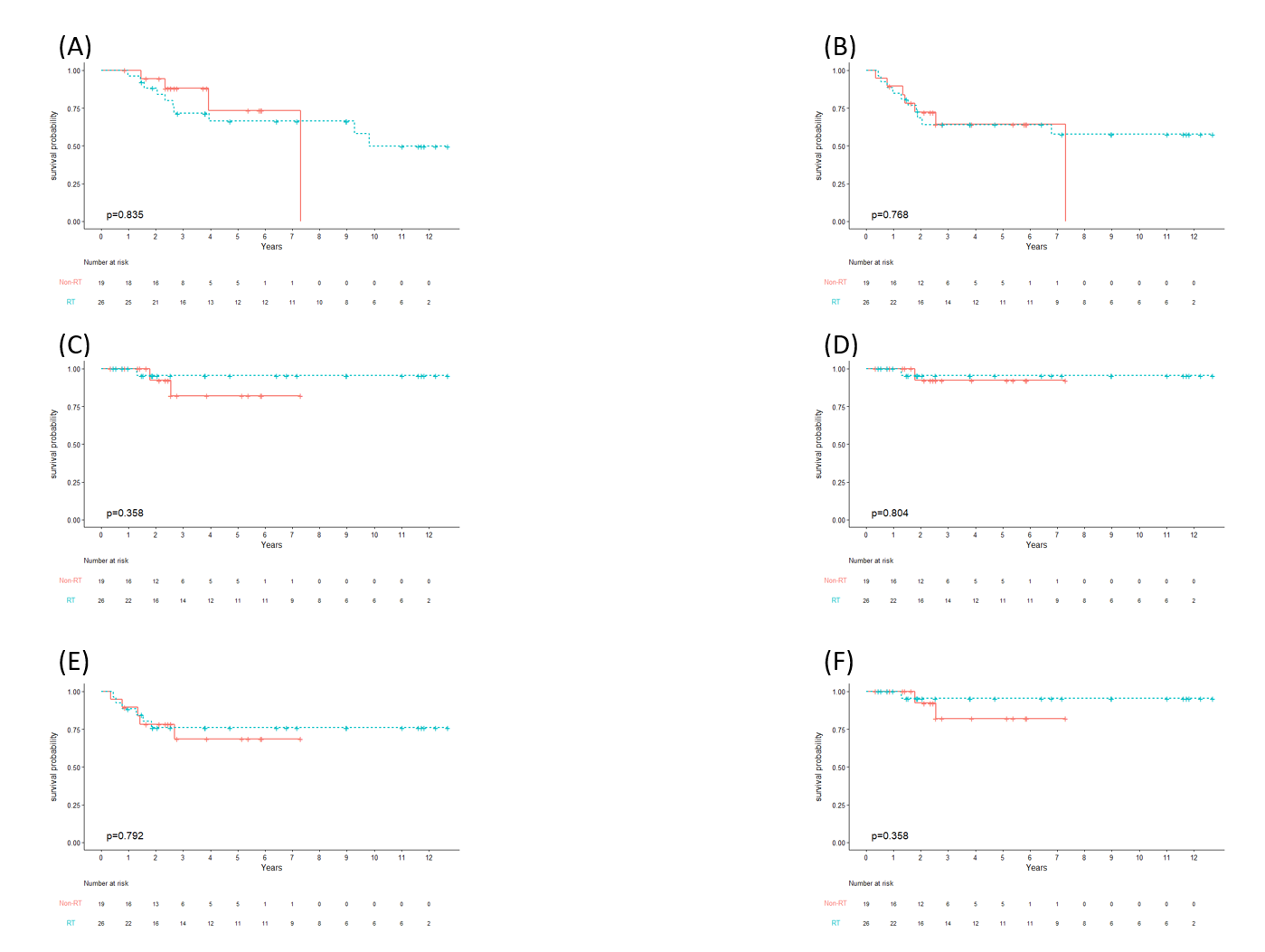


**FIGURE S8.** Kaplan-Meier Plots showing the results of 45 (CCRT: 26; CTx: 19) moderately high risk (T3N1, T4N0, T1N2, T2N2) middle and low rectal adenocarcinoma patients (A) Overall survival; (B) Disease progression-free survival; (C) Local relapse-free survival; (D) Regional relapse-free survival; (E) Distant metastasis-free survival; (F) Locoregional relapse-free survival. RT, radiotherapy; CCRT, concurrent chemoradiation; CTx, chemotherapy.


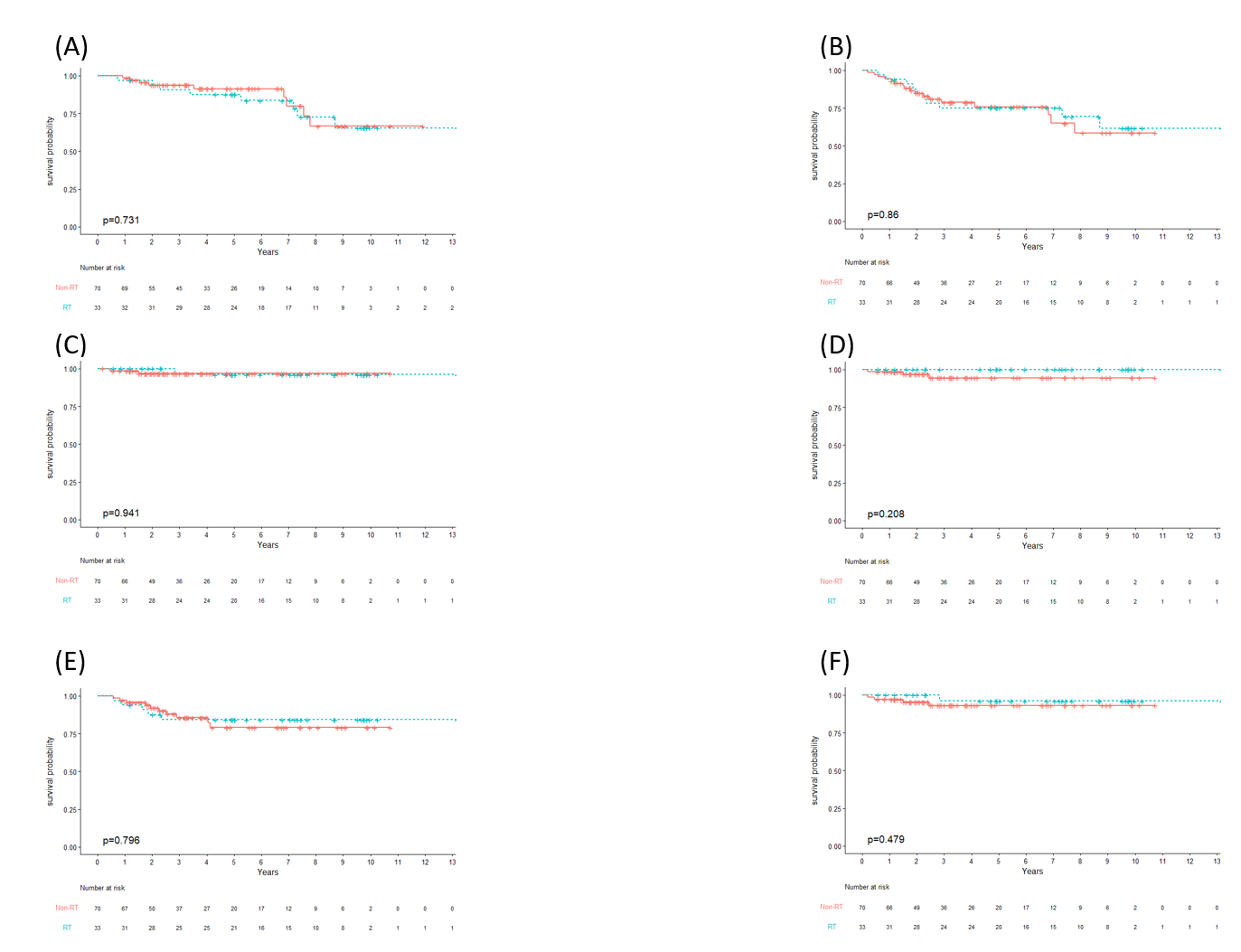


**FIGURE S9.** Kaplan-Meier Plots showing the results of 103 (CCRT: 33; CTx: 70) moderately risk (T1~2N1, T3N0) rectal adenocarcinoma patients (A) Overall survival; (B) Disease progression-free survival; (C) Local relapse-free survival; (D) Regional relapse-free survival; (E) Distant metastasis-free survival; (F) Locoregional relapse-free survival. RT, radiotherapy; CCRT, concurrent chemoradiation; CTx, chemotherapy.


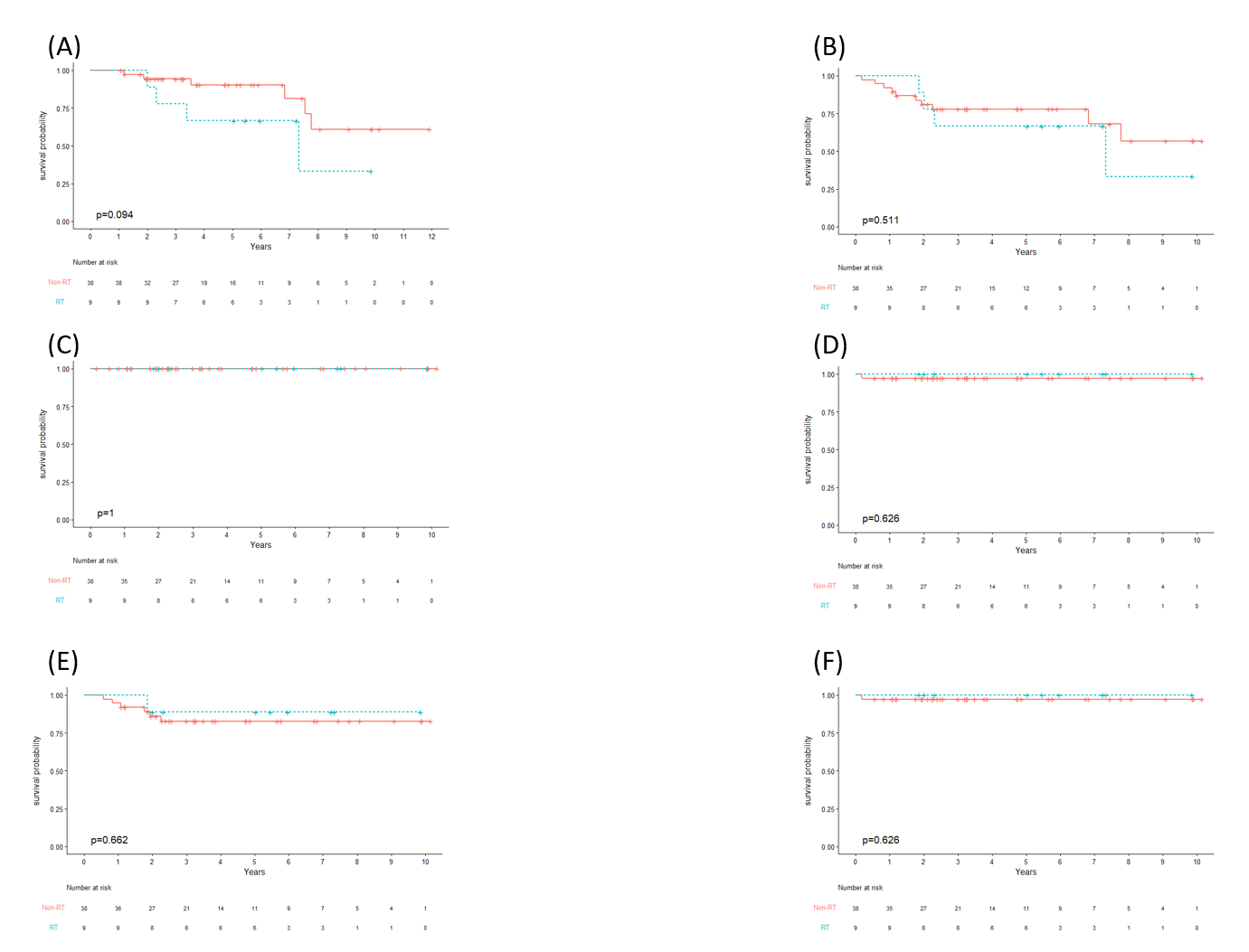


**FIGURE S10.** Kaplan-Meier Plots showing the results of 47 (CCRT: 9; CTx: 38) moderately risk (T1~2N1, T3N0) proximal rectal adenocarcinoma patients (A) Overall survival; (B) Disease progression-free survival; (C) Local relapse-free survival; (D) Regional relapse-free survival; (E) Distant metastasis-free survival; (F) Locoregional relapse-free survival. RT, radiotherapy; CCRT, concurrent chemoradiation; CTx, chemotherapy.


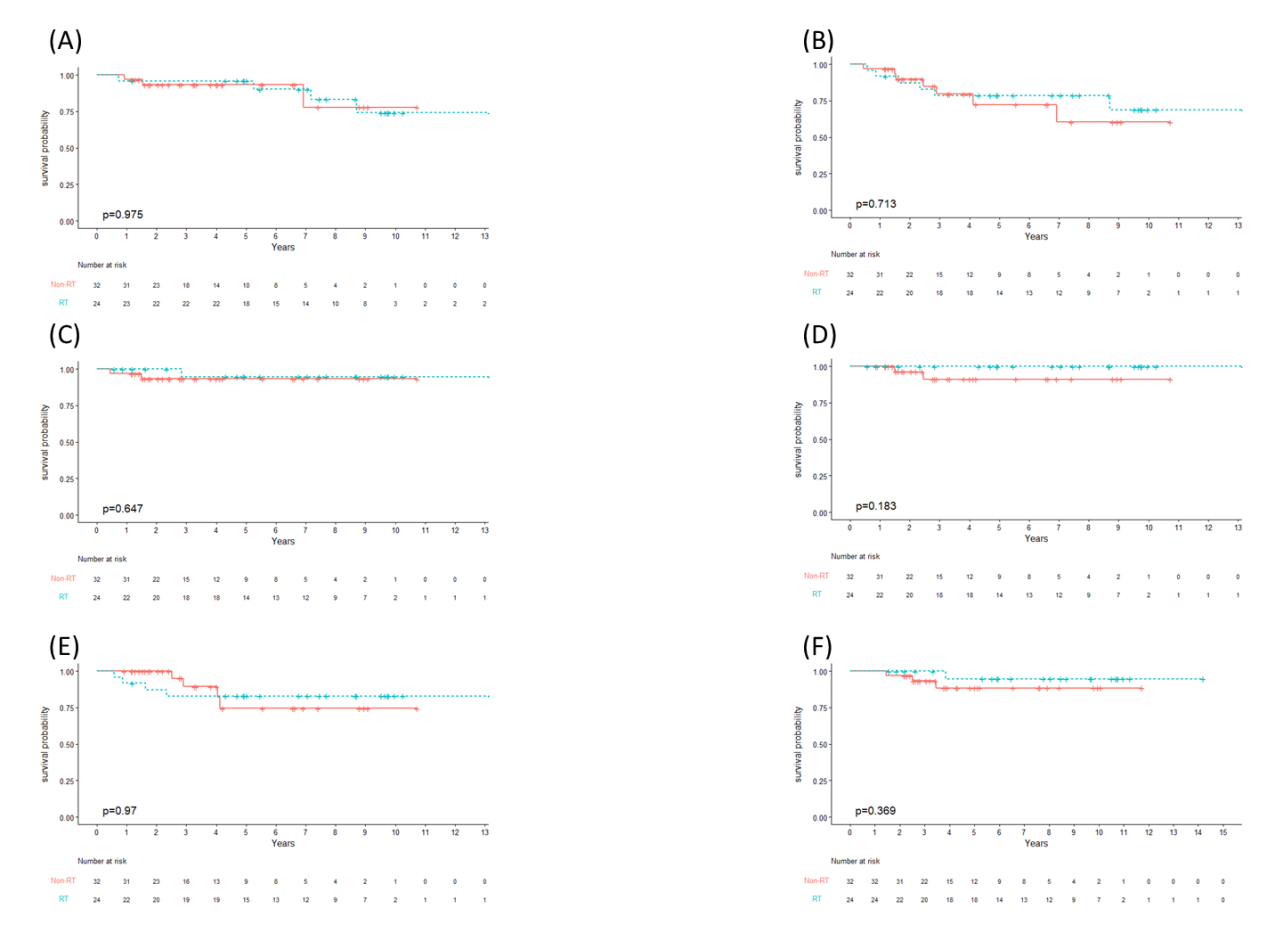


**FIGURE S11.** Kaplan-Meier Plots showing the results of 56 (CCRT: 24; CTx: 32) moderately risk (T1~2N1, T3N0) middle and low rectal adenocarcinoma patients (A) Overall survival; (B) Disease progression-free survival; (C) Local relapse-free survival; (D) Regional relapse-free survival; (E) Distant metastasis-free survival; (F) Locoregional relapse-free survival. RT, radiotherapy; CCRT, concurrent chemoradiation; CTx, chemotherapy.


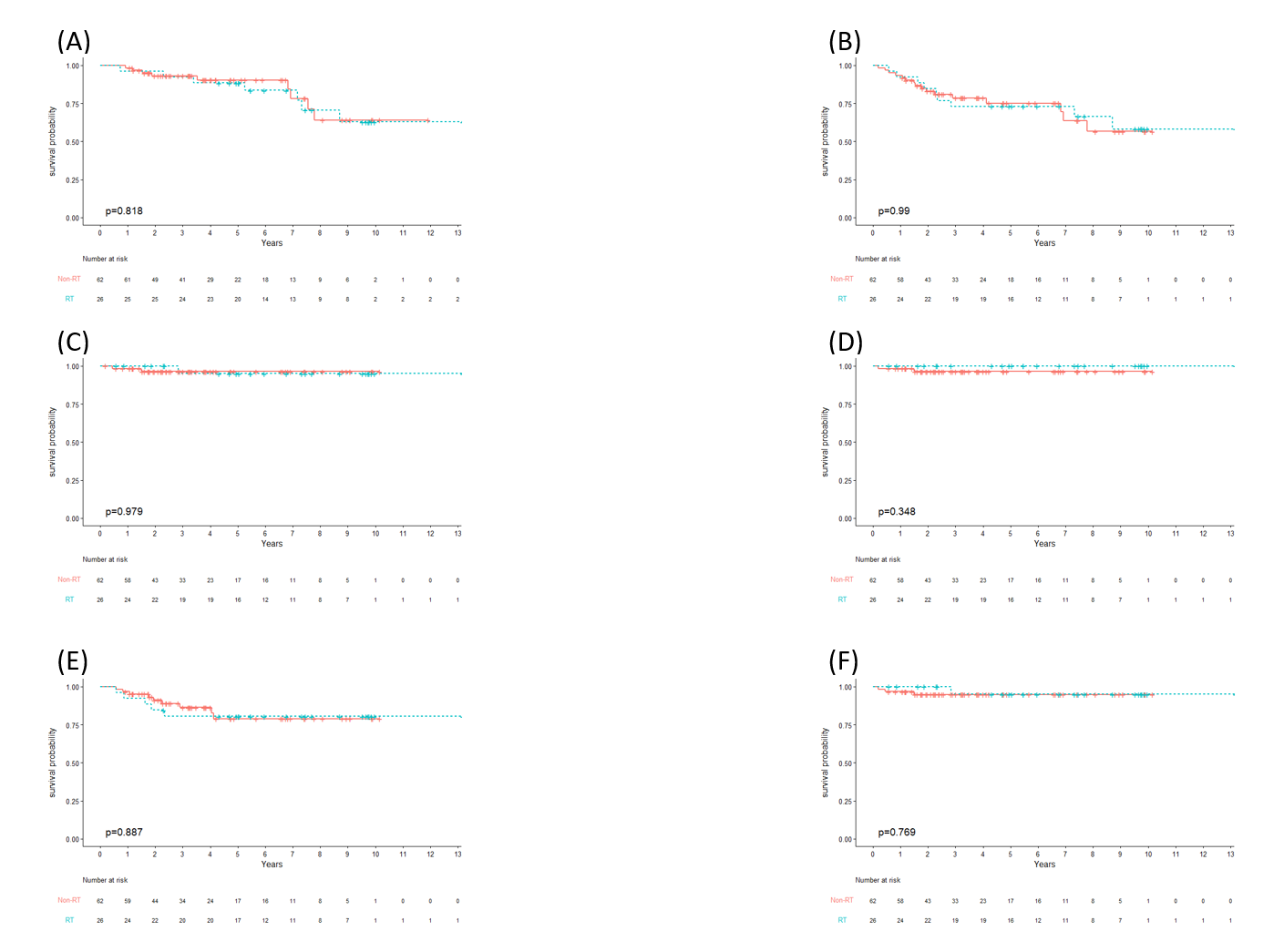


**FIGURE S12.** Kaplan-Meier Plots with the number at risk included showing the results of 88 (CCRT: 26; CTx: 62) pT3N0 rectal adenocarcinoma patients (**A**) Overall survival; (**B**) Disease progression-free survival; (**C**) Local relapse-free survival; (**D**) Regional relapse-free survival; (**E**) Distant metastasis-free survival; (**F**) Locoregional relapse-free survival. RT, radiotherapy; CCRT, concurrent chemoradiation; CTx, chemotherapy.


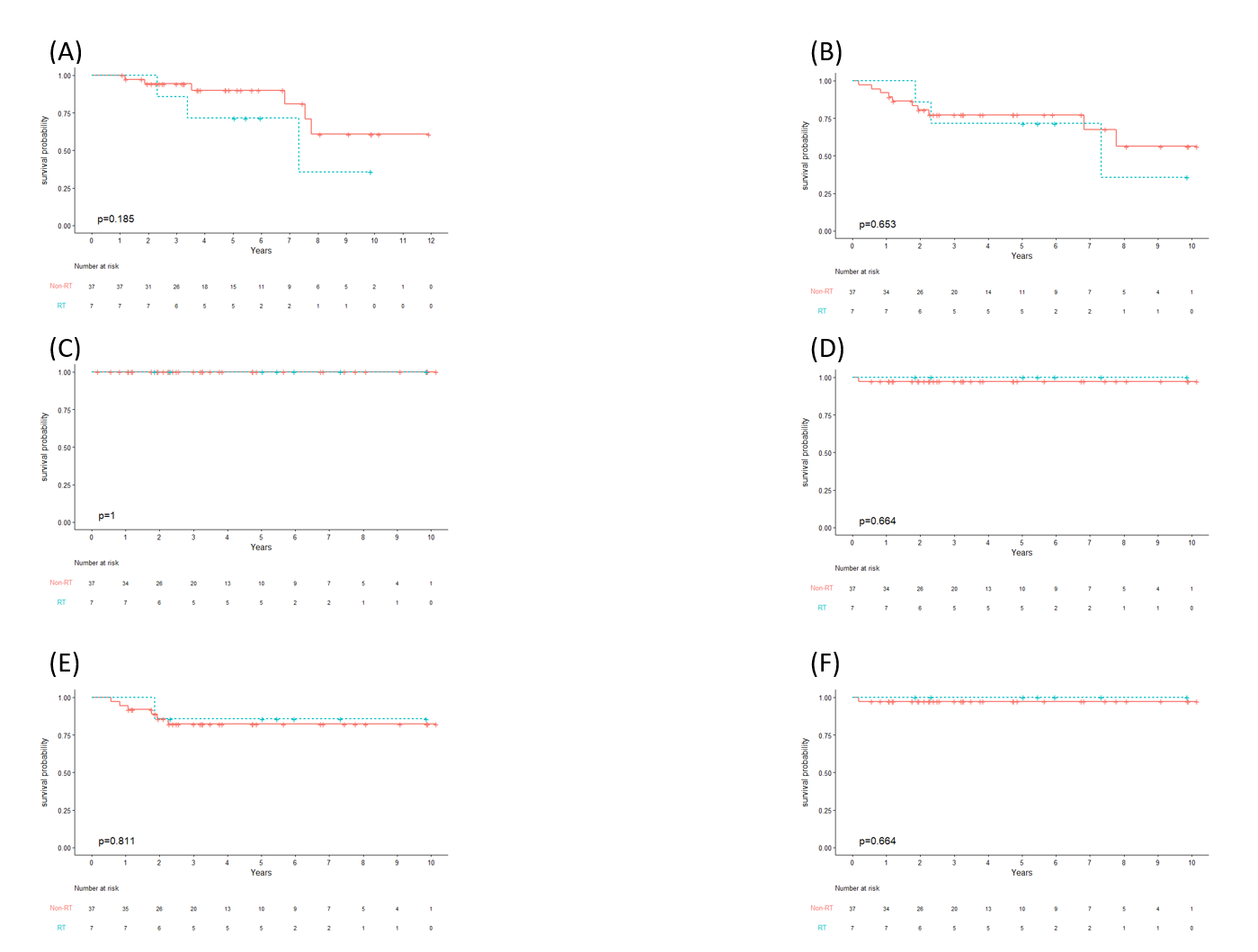


**FIGURE S13.** Kaplan-Meier Plots with the number at risk included showing the results of **44** (CCRT: 7; CTx: 37) proximal pT3N0 rectal adenocarcinoma patients (**a**) Overall survival; (**b**) Disease progression-free survival; (**c**) Local relapse-free survival; (**d**) Regional relapse-free survival; (**e**) Distant metastasis-free survival; (**f**) Locoregional relapse-free survival. RT, radiotherapy; CCRT, concurrent chemoradiation; CTx, chemotherapy.


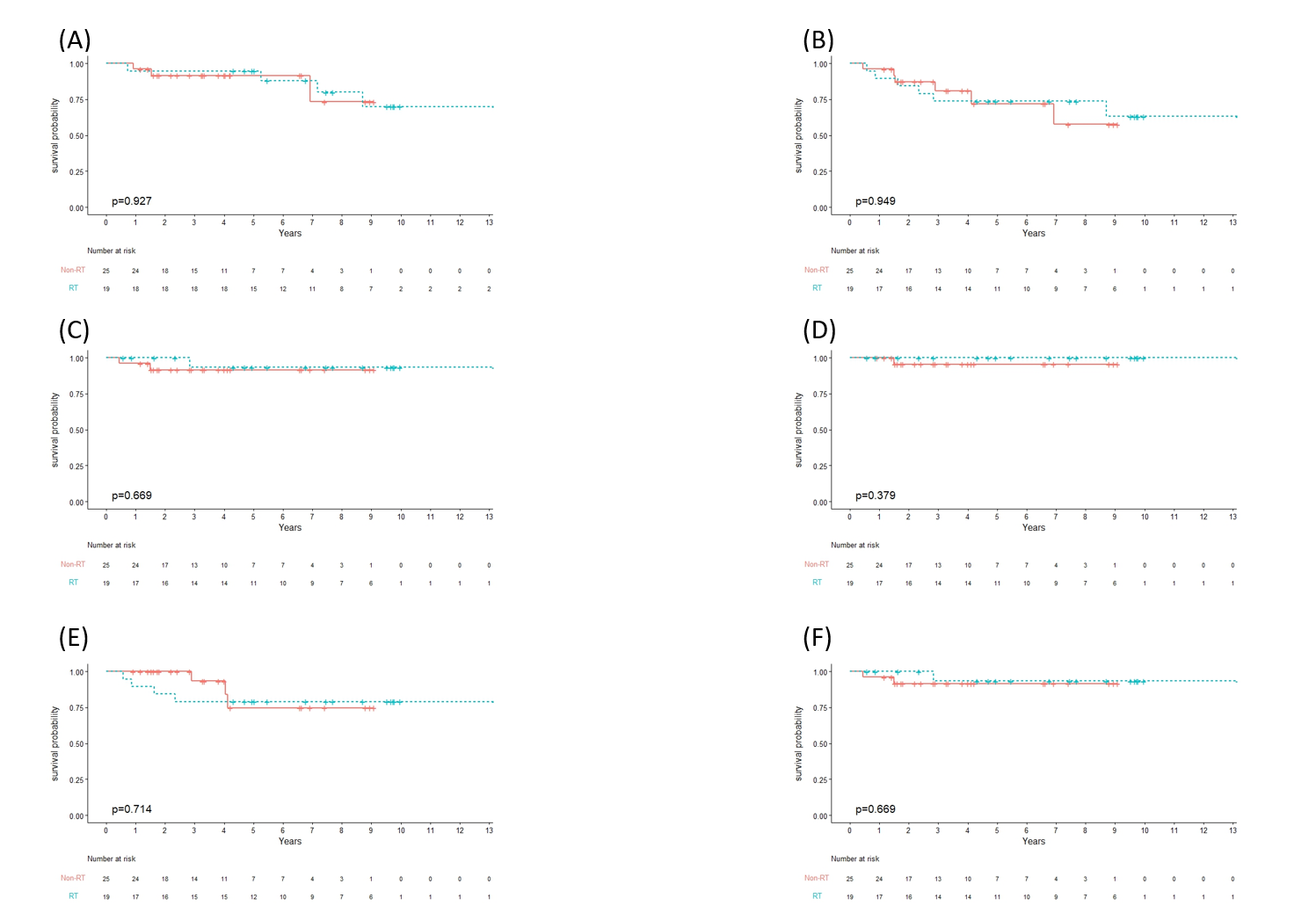


**FIGURE S14.** Kaplan-Meier Plots showing the results of **44** (19 CCRT:; 25 CTx:) middle and low pT3N0 rectal adenocarcinoma patients (**a**) Overall survival; (**b**) Disease progression-free survival; (**c**) Local relapse-free survival; (**d**) Regional relapse-free survival; (**e**) Distant metastasis-free survival; (**f**) Locoregional relapse-free survival.

RT, radiotherapy; CCRT, concurrent chemoradiation; CTx, chemotherapy.
