# Supplementary material for: The impact of intensity‐modulated radiotherapy in conjunction with chemotherapy on proximal pT3N0 rectal cancer patients after total mesorectum excision
Source: Cancer Med. 2023 Nov 6;12(23):21209–18. doi: 10.1002/cam4.6691 (PMC10726884; doi:10.1002/cam4.6691)
Supplement: Supplementary file 2 — Tables S1–S10 [file CAM4-12-21209-s001.docx]

**SUPPLEMENT TABLES**

**TABLE S1.** Clinical and demographic characteristics of the patients in our cohort with pT3N0 rectal cancer

|  |  | CTx only  (n = 62) | % | CCRT  (n = 26) | % | P value |
| --- | --- | --- | --- | --- | --- | --- |
| Sex | Female | 40 | 64.5 | 21 | 80.8 | 0.21 |
|  | Male | 22 | 35.5 | 5 | 19.23 |  |
| Age |  | 67.0±10.2 |  | 61.7±11.7 |  | 0.04 |
| Location | proximal | 37 | 59.7 | 7 | 26.9 | 0.01 |
|  | distal | 25 | 40.3 | 19 | 73.1 |  |
| Resection | LAR | 61 | 98.4 | 24 | 92.3 | 0.43 |
|  | APR | 1 | 1.6 | 2 | 7.7 |  |
| Differentiation | Well | 1 | 1.6 | 0 | 0 | 0.235 |
|  | Moderate | 60 | 96.8 | 24 | 92.3 |  |
|  | Poor | 1 | 1.6 | 2 | 7.7 |  |
| LVI | No | 29 | 46.8 | 11 | 42.3 | 0.88 |
|  | Yes | 33 | 53.2 | 15 | 57.7 |  |
| PNI | No | 49 | 79.0 | 21 | 80.8 | 1 |
|  | Yes | 13 | 21.0 | 5 | 19.2 |  |
| CEA† | <5 | 43 | 72.9 | 17 | 68.0 | 0.85 |
|  | ≥5 | 16 | 27.1 | 8 | 32 |  |
| Size in mm^†^ |  | 51.5±18.2 |  | 48.1±18.5 |  | 0.42 |
| Perforation | No | 60 | 96.8 | 24 | 92.3 | 0.72 |
|  | Yes | 2 | 3.2 | 2 | 7.7 |  |
| Obstruction | No | 42 | 67.7 | 19 | 73.1 | 0.81 |
|  | Yes | 20 | 32.3 | 7 | 26.9 |  |
| Comorbidity | No | 19 | 30.7 | 6 | 23.1 | 0.65 |
|  | Yes | 43 | 69.4 | 20 | 76.9 |  |
| ECOG score | <2 | 57 | 91.9 | 23 | 88.5 | 0.91 |
|  | ≥2 | 5 | 8.1 | 3 | 11.5 |  |

Abbreviations: APR: abdominoperineal resection; LAR: low anterior resection; LVI: lymphovascular invasion; PNI: perineural invasion; LN: lymph node; CTx: chemotherapy; CCRT: concurrent chemoradiation; IV: intravenous; CEA: initial serum carcinoembryonic antigen level; ECOG: initial Eastern Cooperative Oncology Group Performance Status score. ^†^mean ± standard deviation.

**TABLE** S2. Details of 12 first locoregional recurrence cases

|  | Location | AAV cm | P stage | Gunderson's risk | Additional risk | Recurrence months after surgery | RT | Recurrence pattern | Recurrence detail | Lateral nodes mapping | Agree to initial tumor side | Alive | More survival months^†^ | Final condition |
| --- | --- | --- | --- | --- | --- | --- | --- | --- | --- | --- | --- | --- | --- | --- |
| 1 | Middle | 8 | T3N0 | 1 | Perforation | 8 | No | AR | Anastomosis | N/A | N/A | No | 6 | Dead |
| 2 | Middle | 10 | T3N0 | 1 | LVI | 36 | Yes | AR | Anastomosis extending  to right ureter | N/A | N/A | No | 58 | Dead |
| 3 | Middle | 10 | T3N1b | 2 | LVI, PNI | 15 | No | AR | Anastomosis extending  to vagina | N/A | N/A | Yes | 63 | NER |
| 4 | Upper | 10  (RSJ) | T3N1b | 2 | LVI, PNI | 13 | No | AR | Anastomosis | N/A | N/A | No | 40 | Dead |
| 5 | Middle | 6 | T1N2b | 2 | LVI, severe adhesion,  tumor deposits | 18 | Yes | AR+RR [+DM (simultaneously)] | Anastomosis +  pelvic presacral space | N/A. | N/A | No | 2 | Dead |
| 6 | Low | 5 | T3N0 | 1 | LVI, PNI | 21 | No | AR+RR | Anastomosis +  perirrectal nodes | N/A. | N/A | No | 21 | Dead |
| 7 | Low | 3 | T3N1a | 2 | LVI | 24 | No | AR+RR | Anastomosis  left perirectal  obturator node | Obturator | Yes | Yes | 39 | PD under Ctx |
| 8 | Upper | 15 (RSJ) | T3N0 | 1 | Nil | 5 | No | RR (2nd cancer) | Sigmoid colon 2nd cancer (40 cm AAV) | N/A. | N/A | No | 20 | Dead |
| 9 | Middle | 6 | T3N2 | 3 | Nil | 69 | No | RR | Presacral + bilateral  lateral pelvis  confluently LAPs | N/A. | N/A | No | 14 | Dead |
| 10 | Upper | 10  (RSJ) | T3N2b | 3 | LVI, PNI, P-D,  deposit,  very close to serosa | 6 | No | RR [+DM (simultaneously)] | Left perirectal + external  iliac nodes | External iliac | Yes | No | 8 | Dead |
| 11 | Low | 1 | T3N2b | 3 | LVI, P-D, perforation | 17 | Yes | RR | Left ext. iliac node  ( covered by 5040 cGy) | External iliac | Yes | No | 24 | Dead |
| 12 | Low | 5 | T2N1a | 1 | LVI | 32 | No | RR | LAPs over  bil sup  presacral  right external internal  iliac inguinal | Sup presacral  external iliac  internal iliac  obturator inguinal | N/A | No | 45 | Dead |

^†^Data was updated till Sept. 2023; abbreviations: RSJ: rectosigmoid junction; AAV: above anal verge; LVI: lymphovascular invasion; PNI: perineural invasion, P-D: poorly differentiated; RT: radiotherapy; AR: anastomosis recurrence (= local recurrence); RR: regional recurrence; DM: distant metastasis; Bil: bilateral; Sup: superior; N/A: not applicable; Ctx: chemotherapy; PD: progressive disease.

**TABLE S3.** Locoregional and distant relapses in distal rectal cancer patients. Recurrence frequency, site of the first recurrence, and the first salvage treatment modality are shown

|  | Adjuvant CTx only | |  | | Adjuvant CCRT | |  |  |
| --- | --- | --- | --- | --- | --- | --- | --- | --- |
|  | n= 59 | |  | n= 68 | |  | |  |
|  | No. | | % | No. | | % | |  |
| First failure site |  | |  |  | |  | |  |
| Local | 2 | | 3.4 | 1 | | 1.5 | |  |
| Regional | 0 | | 0 | 0 | | 0 | |  |
| Local + regional | 0 | | 0 | 0 | | 0 | |  |
| Locoregional + distant | 4 | | 6.8 | 2 | | 2.9 | |  |
| Distant | 8 | | 13.6 | 18 | | 26.5 | |  |
| First treatment modality for locoregional relapse | |  | | | |  | |  |
| OP + CTx | 0 | | 0 | 0 | | 0 | |  |
| RT+ CTx | 1 | | 16.7 | 0 | | 0 | |  |
| CTx | 2 | | 33.3 | 1^†^ | | 33.3 | |  |
| OP | 0 | | 0 | 0 | | 0 | |  |
| OP + RT | 1 | | 16.7 | 0 | | 0 | |  |
| Nil | 2 | | 33.3 | 2 | | 66.7 | |  |

Abbreviations: CTx: chemotherapy; CCRT: concurrent chemoradiation.

**TABLE S4.** Locoregional and distant relapse in proximal rectal cancer patients. Recurrence frequency, site of the first recurrence, and the first salvage treatment modality are shown

|  | Adjuvant CTx only |  | Adjuvant CCRT |  |
| --- | --- | --- | --- | --- |
|  | n= 76 |  | n= 33 |  |
|  | No. | % | No. | % |
| First failure site |  |  |  |  |
| Local | 1 | 1.3 | 0 | 0 |
| Regional | 1^†^ | 1.3 | 0 | 0 |
| Regional+ distant | 1 | 1.3 | 0 |  |
| Distant | 12 ^‡^ | 15.8 | 9 | 27.2 |
| First treatment modality for locoregional relapse |  |  |  |  |
| OP + CTx | 1 | 33.3 | 0 | 0 |
| RT+ CTx | 0 | 0 | 0 | 0 |
| CTx | 0 | 0 | 0 | 0 |
| OP | 1 | 33.3 | 0 | 0 |
| OP + RT | 0 | 0 | 0 | 0 |
| Nil | 1 | 33.3 | 0 | 0 |

Abbreviations: CTx: chemotherapy; CCRT: concurrent chemoradiation; ^†^ a second cancer over the pelvic sigmoid colon.

**TABLE** S5. Side effects of two arms in 88 pT3N0 cases

|  | Control | Case | p |
| --- | --- | --- | --- |
| Number | 62 | 26 |  |
| Acute |  |  |  |
| Acute diarrhea | 1 (1.64) | 4 (14.81) | 0.041 |
| Acute G3 Hematologic | 3 (4.92) | 2 (7.41) | 0.982 |
| Acute G3 skin | 0 (0.00) | 1 (3.70) | 0.652 |
| Acute G3 any | 16 (25.81) | 12 (46.15) | 0.105 |
| Long-term |  |  |  |
| Long-term G3 GI | 3 (4.92) | 2 (7.41) | 0.982 |
| Long-term G3 stricture anastomsis | 0 (0.00) | 1 (3.70) | 0.652 |
| Long-term G3 U bladder | 0 (0.00) | 1 (3.70) | 0.652 |
| Long-term G3 any | 7 (11.48) | 5 (18.52) | 0.516 |
| 2nd cancer grade |  |  |  |
| 3 | 0 (0.00) | 0 (0.00) | NA |
| 4 | 2 (3.28) | 0 (0.00) | 0.887 |
| 5 | 1 (1.64) | 0 (0.00) | 1.000 |

Abbreviations: G3: grade 3; GI: gastrointestinal.

**TABLE** S6. Univariate and multivariate Cox regression analysis for overall survival in whole 236 patients

|  | Crude HR (95% CI) | p | Adjusted HR (95% CI) | p |
| --- | --- | --- | --- | --- |
| RT + CTx (reference: CTx alone) | 1.27 (0.76 - 2.12) | 0.355 | 0.85 (0.44 - 1.64) | 0.627 |
| Male (reference: female) | 0.84 (0.50 - 1.41) | 0.510 | 0.80 (0.44 - 1.46) | 0.462 |
| Age | 1.05 (1.02 - 1.07) | 0.000 | 1.06 (1.03 - 1.10) | 0.000 |
| Distal rectum (reference: proximal) | 1.08 (0.66 - 1.78) | 0.759 | 1.71 (0.92 - 3.20) | 0.091 |
| APR (reference: LAR) | 1.41 (0.34 - 5.78) | 0.633 | 0.04 (0.00 - 0.60) | 0.019 |
| Poorly differentiated | 2.14 (1.18 - 3.87) | 0.012 | 1.94 (0.81 - 4.65) | 0.136 |
| Lymphovascular invasion (reference: no) | 1.39 (0.71 - 2.73) | 0.341 | 1.13 (0.44 - 2.86) | 0.801 |
| Perineural invasion (reference: no) | 2.14 (1.30 - 3.54) | 0.003 | 1.76 (0.90 - 3.44) | 0.098 |
| CEA ≥5 (reference: <5) | 1.48 (0.88 - 2.48) | 0.136 | 1.52 (0.78 - 2.96) | 0.218 |
| Maximal size | 1.01 (0.99 - 1.02) | 0.386 | 1.00 (0.98 - 1.02) | 0.883 |
| Perforation (reference: no) | 4.23 (1.82 - 9.86) | 0.001 | 6.36 (1.05 - 38.45) | 0.044 |
| Obstruction (reference: no) | 1.98 (1.20 - 3.26) | 0.008 | 1.45 (0.77 - 2.72) | 0.245 |
| Comorbidity (reference: no) | 1.13 (0.65 - 1.97) | 0.653 | 0.64 (0.34 - 1.21) | 0.167 |
| ECOG score ≥2 (reference: <2) | 1.81 (0.94 - 3.47) | 0.076 | 1.51 (0.60 - 3.82) | 0.382 |
| Lymph node involvement ratio | 7.81 (3.10 - 19.70) | < 0.001 | 13.72 (3.43 - 54.93) | < 0.001 |
| T3, 4 (reference: T1, 2) | 1.66 (0.66 - 4.14) | 0.279 | 0.97 (0.34 - 2.75) | 0.951 |

Abbreviations: CTx: chemotherapy; IV: intravenous; RT: radiotherapy; HR: hazard ratio; CI: confidence interval.; APR: abdominoperineal resection; LAR: low anterior resection; CRM: circumferential resection margin; LN: lymph node; IV: intravenous; CEA: initial serum carcinoembryonic antigen level; comorbidity: common comorbidities include chronic diseases involving the circulation, nervous, respiratory, digestive, renal, or the metabolism such as hypertension and diabetes; ECOG score: initial Eastern Cooperative Oncology Group Performance Status score.

**TABLE** S7. Univariate and multivariate Cox regression analysis for disease progression-free survival in whole 236 patients

|  | Crude HR (95% CI) | p | Adjusted HR (95% CI) | p |
| --- | --- | --- | --- | --- |
| RT + CTx (reference: CTx alone) | 1.14 (0.74 - 1.77) | 0.550 | 0.82 (0.48 - 1.41) | 0.472 |
| Male (reference: female) | 1.02 (0.65 - 1.59) | 0.939 | 1.05 (0.64 - 1.71) | 0.845 |
| Age | 1.03 (1.01 - 1.05) | 0.007 | 1.04 (1.01 - 1.06) | 0.002 |
| Distal rectum (reference: proximal) | 1.07 (0.69 - 1.65) | 0.773 | 1.52 (0.91 - 2.53) | 0.111 |
| APR (reference: LAR) | 1.56 (0.49 - 4.95) | 0.449 | 0.48 (0.09 - 2.62) | 0.399 |
| Poorly differentiated | 1.63 (0.88 - 3.01) | 0.117 | 1.17 (0.51 - 2.72) | 0.708 |
| Lymphovascular invasion (reference: no) | 1.48 (0.82 - 2.67) | 0.197 | 1.11 (0.53 - 2.31) | 0.782 |
| Perineural invasion (reference: no) | 1.79 (1.16 - 2.77) | 0.008 | 1.46 (0.84 - 2.53) | 0.177 |
| CEA ≥5 (reference: <5) | 1.49 (0.95 - 2.35) | 0.085 | 1.50 (0.88 - 2.56) | 0.140 |
| Maximal size | 0.99 (0.98 - 1.01) | 0.323 | 0.99 (0.97 - 1.00) | 0.100 |
| Perforation (reference: no) | 2.88 (1.25 - 6.63) | 0.0127 | 4.87 (1.18 - 20.22) | 0.029 |
| Obstruction (reference: no) | 1.42 (0.90 - 2.23) | 0.133 | 1.39 (0.79 - 2.42) | 0.250 |
| Comorbidity (reference: no) | 1.20 (0.735 - 1.96) | 0.467 | 0.82 (0.47 - 1.43) | 0.479 |
| ECOG score ≥2 (reference: <2) | 1.23 (0.66 - 2.26) | 0.515 | 1.06 (0.49 - 2.27) | 0.888 |
| Lymph node involvement ratio | 6.48 (2.85 - 14.7) | < 0.001 | 6.35 (2.01 - 20.09) | 0.002 |
| T3, 4 (reference: T1, 2) | 1.54 (0.71 - 3.34) | 0.277 | 1.15 (0.49 - 2.71) | 0.741 |

Abbreviations: CTx: chemotherapy; RT: radiotherapy; HR: hazard ratio; CI: confidence interval; APR: abdominoperineal resection; LAR: low anterior resection; CRM: circumferential resection margin; LN: lymph node; IV: intravenous; CEA: initial serum carcinoembryonic antigen level; comorbidity: common comorbidities include chronic diseases involving the circulation, nervous, respiratory, digestive, renal, or the metabolism such as hypertension and diabetes; ECOG score: initial Eastern Cooperative Oncology Group Performance Status score.

**TABLE** S8. Univariate and multivariate Cox regression analysis for distant metastasis free-survival in whole 236 cohort patients

|  | Crude HR (95% CI) | p | Adjusted HR (95% CI) | p |
| --- | --- | --- | --- | --- |
| RT + CTx (reference: CTx alone) | 1.45 (0.85 - 2.48) | 0.176 | 1.05 (0.54 - 2.05) | 0.889 |
| Male (reference: female) | 1.25 (0.73 - 2.14) | 0.422 | 1.47 (0.82 - 2.66) | 0.198 |
| Age | 0.99 (0.97 - 1.01) | 0.499 | 1.02 (0.99 - 1.04) | 0.284 |
| Distal rectum (reference: proximal) | 1.30 (0.76 - 2.24) | 0.344 | 2.01 (1.04 - 3.86) | 0.037 |
| APR (reference: LAR) | 2.24 (0.70 - 7.17) | 0.176 | 1.23 (0.22 - 6.78) | 0.810 |
| Poorly differentiated | 1.48 (0.70 - 3.29) | 0.332 | 0.51 (0.15 - 1.70) | 0.275 |
| Lymphovascular invasion (reference: no) | 2.26 (0.97 - 5.29) | 0.059 | 0.91 (0.35 - 2.38) | 0.852 |
| Perineural invasion (reference: no) | 2.33 (1.36 - 4.00) | 0.002 | 1.45 (0.74 - 2.87) | 0.279 |
| CEA ≥5 (reference: <5) | 1.72 (1.00 - 2.97) | 0.052 | 1.86 (0.98 - 3.54) | 0.058 |
| Maximal size | 0.98 (0.97 - 1.00) | 0.020 | 0.98 (0.96 - 1.00) | 0.028 |
| Perforation (reference: no) | 1.90 (0.59 - 6.08) | 0.282 | 0.94 (0.09 - 9.59) | 0.961 |
| Obstruction (reference: no) | 1.12 (0.62 - 2.01) | 0.703 | 1.67 (0.84 - 3.35) | 0.146 |
| Comorbidity (reference: no) | 0.74 (0.43 - 1.29) | 0.293 | 0.53 (0.28 - 1.01) | 0.055 |
| ECOG score ≥2 (reference: <2) | 0.89 (0.38 - 2.09) | 0.794 | 1.09 (0.38 - 3.09) | 0.873 |
| Lymph node involvement ratio | 12.00 (4.89 - 29.70) | < 0.001 | 15.18 (3.86 - 59.61) | < 0.001 |
| T3, 4 (reference: T1, 2) | 1.37 (0.55 - 3.45) | 0.500 | 1.37 (0.48 - 3.88) | 0.555 |

Abbreviations: CTx: chemotherapy; RT: radiotherapy; HR: hazard ratio; CI: confidence interval; APR: abdominoperineal resection; LAR: low anterior resection; CRM: circumferential resection margin; LN: lymph node; IV: intravenous; CEA: initial serum carcinoembryonic antigen level; comorbidity: common comorbidities include chronic diseases involving the circulation, nervous, respiratory, digestive, renal, or the metabolism such as hypertension and diabetes; ECOG score: initial Eastern Cooperative Oncology Group Performance Status score.

**TABLE** S9. Schoenfeld residuals testing proportional hazards by factors

|  | chisq | df | p |
| --- | --- | --- | --- |
| CCRT (reference: CTx alone) | 0.26 | 1 | 0.609 |
| Male (reference: female) | 0.82 | 1 | 0.366 |
| Age | 1.96 | 1 | 0.161 |
| Distal rectum  (reference: proximal) | 5.89 | 1 | 0.015 |
| APR (reference: LAR) | 0.37 | 1 | 0.545 |
| Poorly differentiated | 0.02 | 1 | 0.895 |
| Lymphovascular invasion  (reference: no) | 4.07 | 1 | 0.044 |
| Perineural invasion  (reference: no) | 0.85 | 1 | 0.358 |
| CEA ≥5 (reference: <5) | 0.74 | 1 | 0.390 |
| Maximal size | 1.02 | 1 | 0.313 |
| Perforation  (reference: no) | 0.01 | 1 | 0.905 |
| Obstruction  (reference: no) | 0.02 | 1 | 0.891 |
| comorbidity  (reference: no) | 0.00 | 1 | 1.000 |
| ECOG score ≥2  (reference: <2) | 2.58 | 1 | 0.108 |
| Lymph node involvement  ratio | 0.90 | 1 | 0.342 |
| T3, 4 (reference: T1,2) | 0.04 | 1 | 0.841 |
| Global | 24.10 | 16 | 0.088 |

Abbreviations: CTx: chemotherapy; CCRT: concurrent chemoradiation;. APR: abdominoperineal resection; LAR: low anterior resection; ECOG score: initial Eastern Cooperative Oncology Group Performance Status score.

**TABLE** S10. Stratified univariate and multivariate Cox regression analyses of locoregional relapse-free survival in rectal adenocarcinoma patients

|  | Crude HR (95% CI) | p | Adjusted HR (95% CI) | p |
| --- | --- | --- | --- | --- |
| CCRT (reference: CTx alone) | 0.41 (0.11-1.50) | 0.177 | 0.21 (0.05-0.92) | 0.038 |
| Male (reference: female) | 0.83 (0.25-2.75) | 0.755 | 0.20 (0.05-0.76) | 0.018 |
| Age | 1.05 (0.99-1.10) | 0.094 | 1.08 (1.02-1.15) | 0.012 |
| APR (reference: LAR) | 3.63 (0.47-28.16) | 0.217 | 1.28 (0.06-25.58) | 0.872 |
| Poorly differentiated | 2.59 (0.78-8.59) | 0.120 | 0.24 (0.03-1.77) | 0.161 |
| Perineural invasion  (reference: no) | 1.74 (0.56-5.39) | 0.339 | 2.66 (0.72-9.79) | 0.142 |
| CEA ≥5 (reference: <5) | 1.20 (0.36-4.00) | 0.763 | 1.61 (0.35-7.48) | 0.543 |
| Maximal size | 1.00 (0.97-1.04) | 0.856 | 0.98 (0.94-1.02) | 0.267 |
| Perforation  (reference: no) | 12.22 (3.28-45.47) | 0.001 | 71.31 (5.50-925.14) | 0.001 |
| Obstruction  (reference: no) | 0.53 (0.12-2.43) | 0.415 | 0.60 (0.12-3.07) | 0.538 |
| comorbidity  (reference: no) | 331816714.64 (0.00-Inf) | 0.998 | 79937416.55 (0.00-Inf) | 0.995 |
| ECOG score ≥2  (reference: <2) | 1.48 (0.32-6.76) | 0.613 | 1.23 (0.19-7.74) | 0.828 |
| Lymph node involvement  ratio | 11.73 (1.69-81.34) | 0.013 | 6.97 (0.54-90.06) | 0.137 |
| T3, 4 (reference: T1,2) | 0.70 (0.15-3.22) | 0.651 | 0.10 (0.02-0.47) | 0.004 |
| Stratify (upper or lower) |  |  |  |  |
| Stratify (lymphovascular invasion) |  |  |  |  |

Abbreviations: CTx: chemotherapy; CCRT: concurrent chemoradiation;. APR: abdominoperineal resection; LAR: low anterior resection; ECOG score: initial Eastern Cooperative Oncology Group Performance Status score; CEA: initial serum carcinoembryonic antigen level.
